# Disruption of the Novel Small Protein RBR7 Leads to Enhanced Plant Resistance to Blast Disease

**DOI:** 10.1186/s12284-023-00660-1

**Published:** 2023-09-21

**Authors:** Hui Shi, Qing Xiong, Zhangjie Zhao, Lian Zhou, Junjie Yin, Xiang Lu, Xuewei Chen, Jing Wang

**Affiliations:** State Key Laboratory of Crop Gene Exploration and Utilization in Southwest China, Sichuan Agricultural University, Chengdu, 611130 Sichuan China

**Keywords:** Rice blast, Resistance, Spontaneous lesion, Small protein, Salicylic acid

## Abstract

Plant disease is a threat to global food security. Breeding crops carrying broad-spectrum resistance loci is an effective way to control infectious disease. Disease-resistant mutants are valuable resources for deciphering the underlying mechanisms of plant immunity and could provide genetic loci to generate disease-resistant crops. Here, we identified a rice mutant, *rbr7* (*rice blast resistance 7*), that confers resistance against different strains of *Magnaporthe oryzae*. Disease-mimicking necrotic lesions started to appear on the leaves of *rbr7* four weeks after sowing. Histochemical analysis revealed reactive oxygen species accumulation and cell death accompanied by spontaneous lesion formation in *rbr7*. Map-based cloning and bulk segregation analysis showed a 2855 bp fragment deletion on chromosome 5, leading to the disruption of the *LOC_Os05g28480*-coding protein. Transgenic *rbr7* complementation plants showed compromised resistance to rice blast, indicating that *LOC_Os05g28480*, or *Rbr7*, regulates the rice immune response. *Rbr7* encodes a small protein of unknown function with 85 amino acids. Transcriptomic analysis revealed that disruption of RBR7 led to the upregulation of genes responding to salicylic acid, systemic acquired resistance and pathogenesis-related genes. Taken together, our findings reveal insights into a novel small protein involved in regulating plant resistance to rice blast and provide a potential target for crop breeding.

**Supplementary Information:**

The online version contains supplementary material available at 10.1186/s12284-023-00660-1.

## Introduction

Rice is a staple food for more than half of the world’s population as well as a model for scientific research on monocotyledons. Deciphering the rice immune response with resistance-altered mutants is important for breeding crops with disease resistance. To defend themselves against infectious pathogens, plants have evolved a complicated innate immune system. During plant-pathogen interaction, many molecular events are activated to reprogram the plant for fighting against the invaders, including reactive oxygen species (ROS) bursts, ion fluxes, hormone accumulation, activation of defence-related genes and cell wall fortification (Jones and Dangl 2006; Tsuda et al. 2009; Ngou et al. 2021; Pruitt et al. 2021; Yuan et al. 2021). However, plant growth is usually hampered when immune response is abnormally activated (Wu et al. 2008; Zhang et al. 2022; Zhu et al. 2020b).

Plant hypersensitive response (HR) is a type of programmed cell death that usually occurs at the invasion site of pathogens to stop their expansion to uninfected areas and trigger systemic acquired resistance (SAR) (Coll et al. 2011; Morel and Dangl 1997; Mur et al. 2007). Even without pathogen infection, stress, insect or mechanical damages, HR-like cell death spots have been found on the bodies of different types of lesion mimic mutants (LMMs). In the past years, various LMMs exhibiting HR-like phenotypes have been identified from Arabidopsis (Donahue et al. 2010; Jabs et al. 1996; Mou et al. 2000; Noutoshi et al. 2006), rice (Mizobuchi et al. 2002; Mori et al. 2007; Zeng et al. 2004; Zhu et al. 2016), wheat (Liu et al. 2021; Wang et al. 2016; Yao et al. 2009) and maize (Gray et al. 2002; Hu et al. 1998; Morris et al. 1998). The genes encoding diverse proteins have been characterized from LMMs, including Spotted Leaf 7 (SPL7) (heat stress transcription factor protein) (Yamanouchi et al. 2002), SPL11 (U-box/armadillo repeat domain with E3 ubiquitin ligase activity) (Zeng et al. 2004), SPL28 (clathrin-associated adaptor protein complex 1) (Qiao et al. 2010), Necrotic Leaf Sheath 1 (NLS1) (CC-NB-LRR-type protein) (Tang et al. 2011), SPL5 (RNA splicing protein) (Chen et al. 2012), SPL3 (Mitogen-activated Protein Kinase Kinase Kinase) (Wang et al. 2015b), Lesion Resembling Disease 6-6 (LRD6-6) (multivesicular bodies-localized AAA ATPase) (Zhu et al. 2016), SPL33 (eEF1A-like protein) (Wang et al. 2017c), SPL29 (UDP-N-acetylglucosamine pyrophosphorylase 1) (Xiao et al. 2018), Early Lesion Leaf 1 (ELL1) (cytochrome P450 monooxygenase) (Cui et al. 2021), and Natural Blight Leaf 3 (OsNBL3) (pentatricopeptide repeat protein) (Qiu et al. 2021). LMMs usually exhibited activated immunity response, enhanced disease resistance, accompanied with alteration in growth, which are valuable materials for studying plant immunity response and disease resistance (Wu et al. 2008; Zhang et al. 2022; Zhu et al. 2020b).

Salicylic acid (SA) is a plant defence hormone that plays critical roles in the immune response (Vlot et al. 2009). Upon pathogen attack, plants accumulate endogenous SA to promote the generation of reactive oxygen intermediates and modulate redox balance (Malamy et al. 1990; Metraux et al. 1990; Yang et al. 2004, 2015). For example, in rice, SA levels are correlated with generalized resistance to blast caused by *Magnaporthe oryzae* (*M. oryzae*), and probenazole (PBZ, a type of SA analogue) treatment enhances plant resistance to rice blast (Silverman et al. 1995; Sakamoto et al. 1999). SA-deficient transgenic rice shows compromised resistance to *M. oryzae* due to increased susceptibility to oxidative damage caused by pathogens (Yang et al. 2004). However, overactivated SA signalling usually leads to HR-like cell death on plants. For example, NONEXPRESSOR OF PATHOGENESIS-RELATED GENES 1 (NPR1) is hypothesized to be a transcription cofactor, contributing to the establishment of SAR, a mechanism of induced defence that is activated throughout a plant after exposure to various elicitors (Cao et al. 1994, 1997; Greenberg et al. 2000; Kumar et al. 2022; Zavaliev et al. 2020). Overexpression of *OsNPR1/NPR1 Homolog 1* (*NH1*) in rice can constitutively activate immune response and HR-like cell death, enhances plant resistance to both rice blast and bacterial blight (Chern et al. 2005).

Here, we identified a *rice blast resistance 7* (*rbr7*) mutant that displayed enhanced resistance to different strains of *M. oryzae*. Four weeks post-sowing, the *rbr7* mutant started to exhibit red‒brown mimic lesion spots on the leaf tips. As the plant developed, the mimic lesion spots expanded to the entire leaves. Histochemical staining with 3,3′-diaminobenzidine (DAB) and trypan blue indicated H_2_O_2_ accumulation and cell death accompanied by spontaneous lesion formation in *rbr7*. Map-based cloning and bulk segregation analysis revealed that *Rbr7* encodes an uncharacterized small protein with only 85 amino acids. RNA sequencing analysis suggested that *Rbr7* mainly regulates the rice defence response by affecting SA-dependent SAR. Collectively, our study identifies a novel rice blast resistance mutant *rbr7* and provides insight into the mechanism of SA-dependent SAR in rice regulated by an uncharacterized small protein.

## Materials and Methods

### Plant Materials and Growth Conditions

The *rbr7* mutant was in the *Japonica* background (*Oryza sativa* cv Kitaake) and isolated from an ethyl methane sulfonate (EMS) mutagenesis population of 5000 individuals. Information about the Kitaake cultivar can be found at the Phytozome website (https://phytozome-next.jgi.doe.gov/info/OsativaKitaake_v3_1). Spontaneous lesions of the *rbr7* mutant were observed in the field approximately four weeks after gemination. Jodan (*Oryza sativa ssp. indica*) was crossed with *rbr7* for map-based cloning (Zhu et al. 2020a). For material phenotypic investigation, crossing and propagation, the plants were grown in the fields of Sichuan Agricultural University in Wenjiang, Chengdu, Sichuan, or in Lingshui, Hainan, China.

### Spray and Punch Inoculation with *M. oryzae*

Isolates of *M. oryzae* Zhong10-8-14, ZE-1 and 0755-1-1 were used for inoculation. For spray inoculation, three-week-old rice plants grown in the field (prior to the appearance of spontaneous lesion spots on *rbr7*, Additional file 1: Fig. S1) were sprayed with blast spore suspensions (in 0.1% Tween-20) at a concentration of 2 × 10^5^ spores/mL. Lesion numbers of the inoculated plant leaves were counted and photographed 6–7 days post-inoculation.

For punch inoculation, three-week-old rice plants were grown in a greenhouse under 16 h/8 h light/dark at 26–28 °C. The fully expanded leaves of the plants were removed, punched, and inoculated with blast spore suspensions (in 0.1% Tween-20) at a concentration of 2 × 10^5^ spores/mL. The inoculated leaves were then placed on the surface of 6-benzylaminopurine (6-BA) solution (1 μg/mL) in a growth chamber with 16 h/8 h light/dark at 28 °C. The lesion length of the inoculated leaves was measured and photographed 6–7 days after inoculation.

### qRT‒PCR

Total RNA was extracted from plant leaf samples using TRIzol™ Reagent (Invitrogen, USA) following the procedures provided by the manufacturer. HiScript III RT SuperMix for qPCR (+ gDNA wiper) (Vazyme, China) was applied to obtain cDNA samples to perform qPCR analysis with QuantiNova™ SYBR^®^ Green (Qiagen, Germany) and a Bio-Rad CFX96™ System coupled to a C1000 Thermal Cycler (Bio-Rad, USA). The reference gene *Ubiquitin* (*UBQ*) (LOC_Os03g13170) was used as an internal control. The primers used are listed in Additional file 3: Table S2.

### Shading Treatment

The leaves of Kitaake and the *rbr7* mutant were partly shaded with aluminium foil prior to the appearance of spontaneous lesion spots on *rbr7* until the presence of mimic lesions on the nonshaded part of the *rbr7* leaves was observed. The aluminium foil was removed, and the leaves were then photographed.

### Trypan Blue and DAB Staining

A trypan blue staining assay was performed on fresh leaves following previous descriptions (Yin et al. 2000). Leaf samples were collected and immersed in trypan blue solution (2.5 mg/mL trypan blue, 25% lactic acid, 23% water-saturated phenol, 25% glycerol), boiled over water for 2 min, and then destained with 30% chloral hydrate solution for three days, during which the solution was changed multiple times. After destaining, the samples were equilibrated within 50% glycerol for five hours, and then photographs were taken.

Detection of H_2_O_2_ accumulation was carried out using a DAB staining assay as described previously (Thordal-Christensen et al. 1997). The samples were submerged in 1 mg/mL DAB containing 10 mM MES (pH 6.5) for 12 h in the dark at 30 °C. Then, the leaf samples were transferred to a solution with 90% ethanol and 10% glycerol and incubated at 90 °C until chlorophyll was completely removed. Cleared leaves were photographed using an Olympus anatomical microscope.

### Genetic Analysis, Map-Based Cloning and Bulk Segregation Analysis Sequencing

Reciprocal crosses were carried out between the *rbr7* mutant and Kitaake for genetic analysis. The F_1_ and F_2_ populations were used for phenotyping to analyse the segregation ratio (Additional file 2: Table S1). The F_1_ populations exhibited Kitaake-like phenotypes. The F_2_ population segregated at a 3 to 1 ratio of Kitaake-like phenotype versus *rbr7* mutant mimic-lesion phenotype.

To determine the locus of *Rbr7*, *rbr7* was crossed with Jodan (*Oryza sativa ssp. indica*), and the F_2_ population derived from the Jodan × *rbr7* cross was used for map-based cloning. We first pooled and applied 6 individuals exhibiting the mutant phenotype with 30 simple sequence repeat (SSR) markers distributed along the 12 chromosomes to determine the linkage site between the SSR marker RM18380 and RM440 located on chromosome 5. Then, we used 44 individuals with the mutant phenotype to confirm that the location of *Rbr7* was between these two markers on chromosome 5. More markers located between RM18380 and RM440 were further developed, and 386 mutant-like individuals from the F_2_ populations were used to localize *Rbr7* to a 1.5 Mb region between the insertion‒deletion (InDel) marker I21 and the SSR marker RM18522. Then, we developed other InDel markers, GM1, GM2 and GM3, to analyse 460 F_2_ mutant-like individuals and mapped the locus for *Rbr7* to a 400-kb interval on chromosome 5 between GM1 and GM2 (Fig. 3A). Primers for the markers used are listed in Additional file 3: Table S2.

Bulk segregation analysis (BSA) sequencing was performed with 20 pooled mutant phenotype individuals and 20 pooled wild-type phenotype individuals from the F_2_ population to provide more evidence for the locus of *Rbr7* at Biomarker Technologies (Wuhan, China). The clean reads were aligned to the reference Nipponbare genome MSU 7.0 (http://rice.uga.edu/) and Kitaake genome v3.1 (https://kitbase.ucdavis.edu/) by using Bowtie2. The BSA sequencing data can be found under the accession number PRJCA019370 in the National Genomics Data Center (https://ngdc.cncb.ac.cn). The PCR duplicates were removed by the Picard tools. Then, SNPs were called by using GATK. Mutated allele frequency was calculated with genotyping depth data resulting from GATK calling by using a custom PERL script. The scatter plot of mutated allele frequency was visualized with Matplotlib in Python. For large deletions, the average sequencing depth was calculated in a 100 bp window with a 5 bp step by using SAMtools. The ratio of *rbr7*/Kitaake was calculated with a custom script. Then, large deletions were filtered out by rbr7/Kitaake < 0.05. Adjacent deletion windows were merged. Deletion of a 2855 bp fragment was detected in the mapped 400-kb region (between GM1 and GM2 on chromosome 5), and there is only one gene, *LOC_Os05g28480* (NCBI accession ID: LOC4338506), located within the deletion fragment.

Primers PF1, PF2 and PR1 (Additional file 3: Table S2) were designed according to the fragment deletion for a cosegregation analysis (Additional file 1: Fig. S3).

### Vector Construction and Rice Transformation

The complementation plasmid pCambia1300-*proRbr7*-*Rbr7* containing the 1531 bp upstream region, the 2472 bp genomic sequence and 1091 bp downstream region of LOC_Os05g28480, covering the whole deletion fragment, was constructed and transformed into the *rbr7* mutant. The sequence was amplified with the primers listed in Additional file 3: Table S2 and recombined into the vector pCambia1300 using the Vazyme ClonExpress^®^ II One Step Cloning Kit (Vazyme, China) following the manufacturer’s description. The complementation plasmid was transformed into the *rbr7* mutant through *Agrobacterium tumefaciens* strain EHA105. Rice transformation was carried out by BioRun (Wuhan, China) as previously described (Wang et al. 2017a).

CRISPR/Cas9-mediated knockout of *Rbr7* was performed by BioRun (Wuhan, China). The knockout plants were confirmed by PCR and sequencing of the target region for detailed mutation type. Primers are listed in Additional file 3: Table S2.

For subcellular localization, the pRTVc-*Rbr7*-GFP construct was generated with the coding sequence of *Rbr7* fused in-frame with GFP. The sequence of *Rbr7* was generated with the primers listed in Additional file 3: Table S2. The PCR product was recombined into the linearized vector pRTVc-GFP.

### Subcellular Localization Assays

To determine the subcellular localization of RBR7 in rice protoplasts, the pRTVc-*Rbr7*-GFP construct was transformed into protoplasts prepared from Kitaake seedlings by polyethylene glycol-mediated transfection as previously described (Bart et al. 2006). As a control, pRTVc-GFP was also transformed under the same conditions. H2B-RFP was cotransformed as a nuclear marker. Fluorescence was examined under a confocal microscope (Leica STELLARIS STED/EM CPD300) 16 h after transformation and incubation at 28 °C under darkness. Total protein of the rice protoplast transformed with pRTVc-GFP and pRTVc-*Rbr7*-GFP was extracted respectively. Immunoblotting was carried out to detect the GFP and RBR7-GFP proteins with anti-GFP rabbit polyclonal antibody (Sangon Biotech, Shanghai, China) as described previously (Wang et al. 2017b).

### Transcriptome Library Construction and Sequencing

Leaves of three-week-old seedlings of the *rbr7* mutant and Kitaake grown in a greenhouse (16 h/8 h light/dark, 26–28 °C) were harvested in liquid nitrogen. Three biological replicates of total RNA were isolated using TRIzol reagent (Invitrogen, USA) according to the manufacturer’s description. RNA quality was checked using RNase-free agarose gel electrophoresis and assessed on an Agilent 2100 Bioanalyzer (Agilent, USA). Strand-specific libraries were constructed with the NEBNext Ultra RNA Library Prep Kit for Illumina (NEB, USA) and then sequenced on an Illumina NovaSeq 6000 sequencer by Novogene (Beijing, China).

The clean reads were mapped to the rice reference genome (MSU 7.0) by HISAT2 (v2.2.1). The sequence data can be found under the accession number PRJCA016897 in the National Genomics Data Center (https://ngdc.cncb.ac.cn). The differentially expressed genes (DEGs) were identified by Cuffdiff (v2.2.1). DEGs were defined as genes with a fold change ≥ 2.0 and an adjusted *p* value ≤ 0.05. A complete list of DEGs is shown in Additional file 6: Table S5. GO enrichment analysis of DEGs was performed by the topGO package.

## Results

### *rbr7* Showed Enhanced Resistance to Rice Blast

**Fig. 1:**
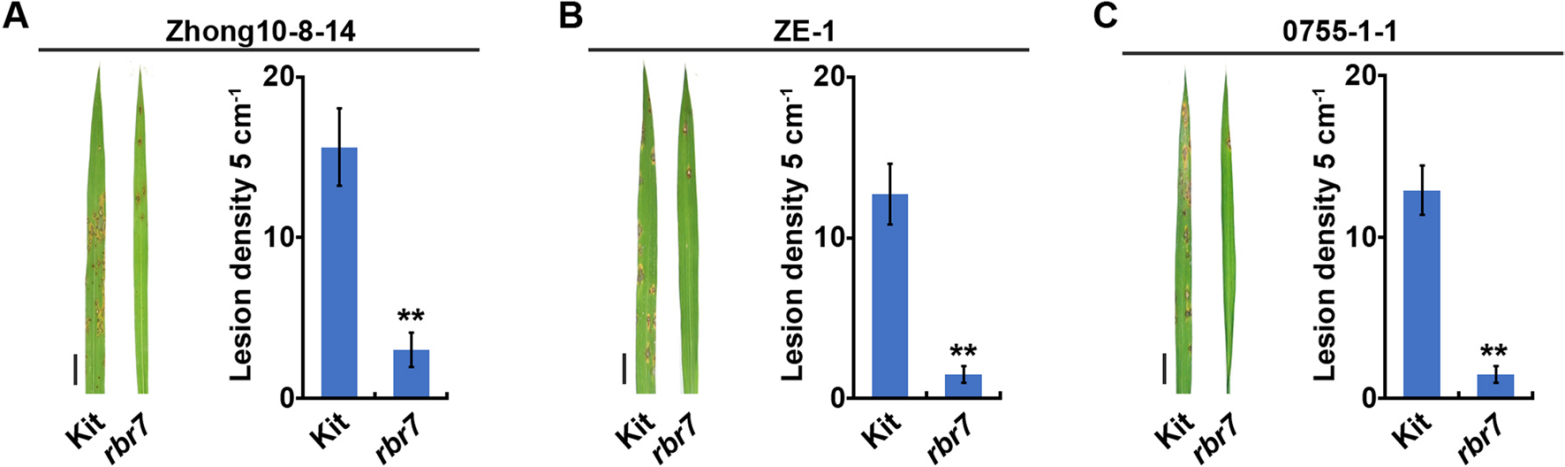
Identification of a rice blast resistance mutant *rbr7*. Spray inoculation with spores of *M. oryzae* isolates Zhong10-8-14 (**A**), ZE-1 (**B**) and 0755-1-1 (**C**). The left panels are leaves of Kit and *rbr7* after inoculation. Bars = 1 cm. The right panels are lesion density per 5 cm on leaves of Kit and *rbr7*. The values are means ± SD of 10 leaves per sample. The asterisks indicate significant difference compared with Kit (*P* < 0.01, student’s *t* test)

To identify rice blast resistance genes, we generated an EMS mutagenesis mutant population containing ∼5000 lines in the rice Kitaake (Kit) cultivar variety. From these mutant lines, we isolated a mutant *rbr7* showing enhanced resistance to *M. oryzae* compared with Kit in the field. We further inoculated *rbr7* with spores derived from different Kitaake-compatible *M. oryzae* isolates Zhong10-8-14, ZE-1 and 0755-1-1 and found that there was lower lesion density on the leaves of *rbr7* than on those of Kit after inoculation (Fig. 1, Additional file 1: Fig. S1).

Next, examination of blast disease resistance by punch inoculation with *M. oryzae* showed that the *rbr7* plants displayed significantly smaller lesions than the Kit plants. Fungal DNA quantification confirmed that the *rbr7* leaves harboured significantly lower *M. oryzae* biomass (less than 50%) than the leaves of wild-type plants, indicating that the *rbr7* mutant showed enhanced resistance to rice blast than Kit (Additional file 1: Fig. S2).

### *rbr7* Harboured a Light-Induced Spotted-Leaf Phenotype

**Fig. 2:**
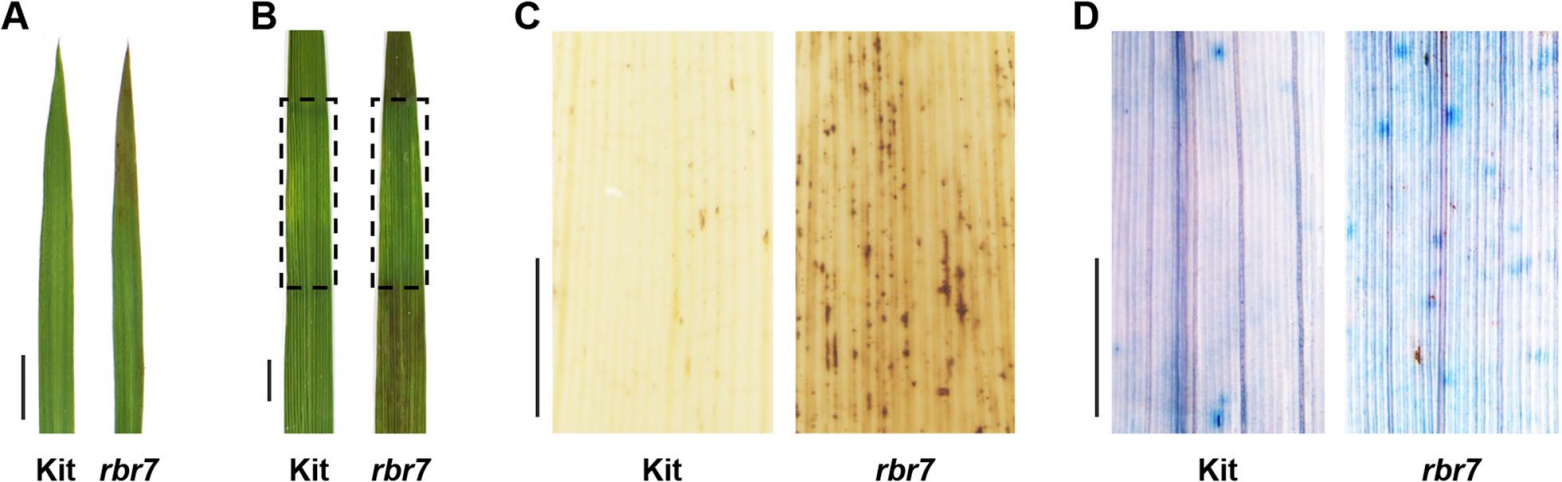
Characterization of the leaf lesion mimic phenotype of *rbr7*. **A** Leaves from Kit and *rbr7* grown in the natural field. Necrotic mimic lesions started to appear on the leaf of *rbr7*. Bar = 1 cm. **B** Leaves of Kit and *rbr7* after shading treatment. The aluminium foil covered part of *rbr7* showed no mimic lesion. The dashed rectangles indicated the covered part. Bar = 1 cm. **C** DAB staining for H_2_O_2_ accumulation. The brown spots represented H_2_O_2_ accumulation on *rbr7* leaves. Bar = 1 mm. **D** Trypan blue staining for cell death. The blue staining indicated cell death on *rbr7* leaves. Bar = 1 mm

When the *rbr7* mutant was grown in the field, spot-like spontaneous lesions appeared on the four-week-old seedlings that initiated on the leaf tips and then spread throughout the entire leaves (Fig. 2A). Previous studies have shown that several environmental factors, such as light, may induce mimic lesion spot formation (Cui et al. 2021; Wang et al. 2015a). We then performed a light-shading experiment to test whether mimic lesion spot formation in the *rbr7* mutant was dependent on light. Compared to the presence of mimic lesions on the unshaded part of the *rbr7* mutant, the shaded part of *rbr7* produced no spontaneous lesion spots (Fig. 2B). These results indicate that the lesion mimic phenotype of *rbr7* is light-induced.

Mimic lesion spot formation is usually accompanied by ROS burst and cell death. We first performed a DAB staining assay to determine the H_2_O_2_ distribution and found that the *rbr7* mutant displayed much stronger staining than Kit (Fig. 2C). Next, we carried out a trypan blue staining assay, which is a traditional method for selectively staining dead tissues or cells. A large number of blue spots were spread on the leaves of *rbr7*, whereas few signals were detected on Kit (Fig. 2D). Taken together, the results of histochemical staining indicated that the *rbr7* mutant exhibited an elevated immune response and possibly the initiation of HR.

### Map-Based Cloning and Bulk Segregation Analysis (BSA) of *rbr7*

To determine whether the *rbr7* mutant phenotype was controlled by a single gene, a backcross of the *rbr7* mutant with Kit was carried out. The F_1_ generation showed a similar phenotype to the wild-type Kit, and the F_2_ generation segregated at a 3 to 1 ratio (wild-type phenotype: *rbr7* mutant phenotype). Moreover, in reciprocal crosses as well as in the cross with Jodan, the F_1_ and F_2_ populations showed consistent results (Additional file 2: Table S1). Taken together, the results suggested that the *rbr7* mutant harbours a recessive mutation controlled by a single gene.

**Fig. 3:**
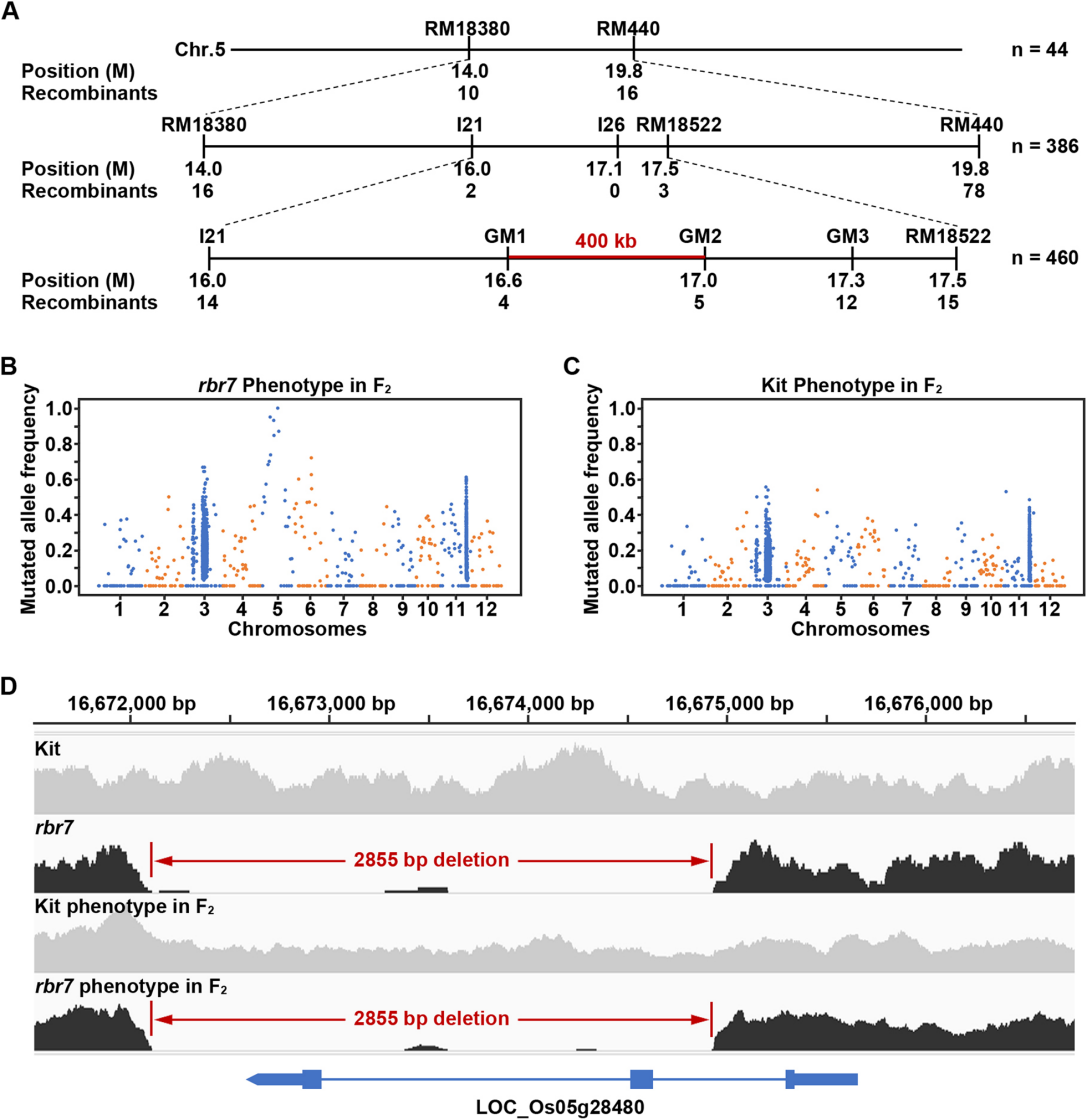
Map-based cloning and bulk segregation analysis of *rbr7*. **A** Map-based cloning of *rbr7*. The *Rbr7* locus was mapped to a 400-kb region between marker GM1 and GM2 on chromosome 5. The numbers below the markers indicate the position and the number of recombinant individuals among the examined individuals with the mutant phenotype from the F_2_ population. **B**, **C** Mutated allele frequency of *rbr7* (**B**) and Kit (**C**) phenotype pool from F_2_ population with bulk segregation analysis sequencing. The scatter plot was visualized with Matplotlib in Python. **D** Coverage plot of Kit, *rbr7*, Kit phenotype pool from F_2_ population and *rbr7* phenotype pool from F_2_ population on chromosome 5. The 2855 bp deletion region was indicated in red

An F_2_ population derived from the Jodan × *rbr7* cross was applied for map-based cloning. Six individuals with the mutant phenotype from the F_2_ generation were pooled and applied together with 30 SSR markers distributed along the 12 chromosomes to determine the linkage site. The SSR markers RM18380 and RM440 located on chromosome 5 were found to be highly linked with the mutant phenotype. Using 44 individuals with the mutant phenotype from the F_2_ generation, we confirmed that the location of *Rbr7* was between these two markers on chromosome 5. More markers located between RM18380 and RM440 were further developed, and 386 mutant-like individuals from the F_2_ populations were used to locate *Rbr7* to a 1.5 Mb region between the InDel marker I21 and the SSR marker RM18522. Then, we developed other InDel markers, GM1, GM2 and GM3, to analyse 460 F_2_ mutant-like individuals. With the help of these three new markers, we delimited the locus for *Rbr7* to a 400-kb interval on chromosome 5 between GM1 and GM2 (Fig. 3A).

Since no markers exhibiting polymorphisms between *rbr7* and Jodan could be found and there were still 67 candidate genes in this region (Additional file 4: Table S3), we carried out bulk segregation analysis sequencing with pooled mutant-like individuals and wild-type-like individuals. A significantly high frequency of mutated alleles was found in the mutant phenotype pool compared with the Kit phenotype pool on chromosome 5, whose location was in accordance with the 400-kb mapped region (Fig. 3B, C). Through large deletions analysis, a 2855 bp fragment deletion was detected within the 400-kb region (Fig. 3D, Additional file 5: Table S4). Primers were designed in and around the fragment for a cosegregation analysis, and the 2855 bp deletion fragment was found to be highly linked to the mutation phenotype in the F_2_ population (Additional file 1: Fig. S3). *LOC_Os05g28480* was the only gene located in the deletion region, suggesting that it might be the candidate gene responsible for the mutation phenotype of *rbr7*.

### Complementation Analysis of *Rbr7*

**Fig. 4:**
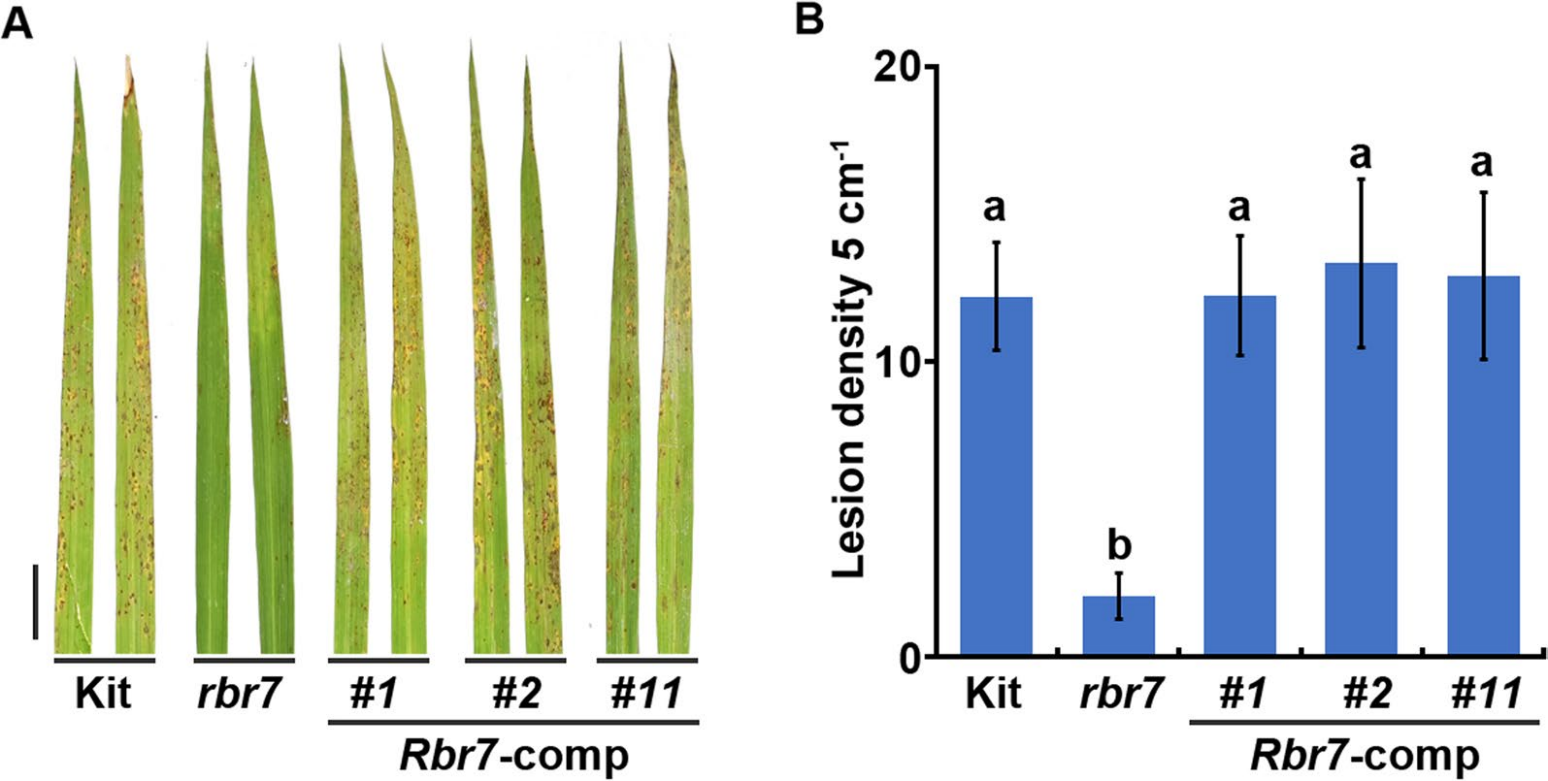
Rice blast resistance phenotype of the complemented lines. **A** Leaves of Kit, *rbr7* and the complemented lines of *rbr7* (*Rbr7*-comp) after spray inoculation with the *M. oryzae* isolate Zhong10-8–14. Three-week-old seedlings were used for spray inoculation. Bar = 1 cm. **B** Lesion density per 5 cm on leaves represented in (A). The values are means ± SD of 10 leaves per sample. Letters above each column indicate significant difference between the compared pairs (*P* < 0.05, one-way ANOVA with Tukey’s test)

To confirm that the 2855 bp deletion did not affect the adjacent genes, qPCR was carried out to analyse the expression levels of *LOC_Os05g28460*, *LOC_Os05g28470* and *LOC_Os05g28500*, and no significant difference was found between Kit and *rbr7* (Additional file 1: Fig. S4). The 2855 bp deletion in *rbr7* led to a deletion from the first intron to the downstream intergenic region of *LOC_Os05g28480*, resulting in a 2082 bp deletion of its genomic sequence and disrupting the protein coding of LOC_Os05g28480. To test whether *LOC_Os05g28480* was the candidate gene for *rbr7*, a plasmid (pCambia1300-*proRbr7*-*Rbr7*) harbouring the 5094 bp sequence of the candidate gene, including the 1531 bp upstream region, 2472 bp genomic region and 1091 bp downstream region (covering the whole deletion fragment), was transformed into the *rbr7* mutant. At the T2 generation, three *Rbr7* complementation (*Rbr7*-comp) lines exhibited similar lesion densities as Kit in the spray inoculation assay with *M. oryzae* isolate Zhong10-8-14 (Fig. 4A). The lesion density showed no significant difference between Kit and the *Rbr7*-comp lines (Fig. 4B). For the punch inoculation assay, the enhanced resistance of *rbr7* was obviously restored to the Kit level in the *Rbr7*-comp lines (Additional file 1: Fig. S5A, B). Moreover, no mimic lesion spots were observed on the leaves of the *Rbr7*-comp plants (Additional file 1: Fig. S5C). However, the *rbr7* mutant exhibited reduced plant height, the *Rbr7*-comp plant had not restored its height to wild-type level (Additional file 1: Fig. S6).

To further validate the genetic role of *RBR7*, we generated knockout lines of *Rbr7* (*Rbr-*KO) using CRISPR/Cas9 technology (Additional file 1: Fig. S7A). Similar as the *rbr7* mutant, there were lots of mimic lesions on the leaves of *Rbr7*-KO plants (Additional file 1: Fig. S7B), and *Rbr-*KO plants showed enhanced resistance than Kit with *M. oryzae* inoculation (Additional file 1: Fig. S7C–F). These results demonstrated that the *LOC_Os05g28480* gene is *Rbr7,* which is responsible for the *rbr7* mutation.

### Expression Pattern of *Rbr7* and Protein Subcellular Localization

**Fig. 5:**
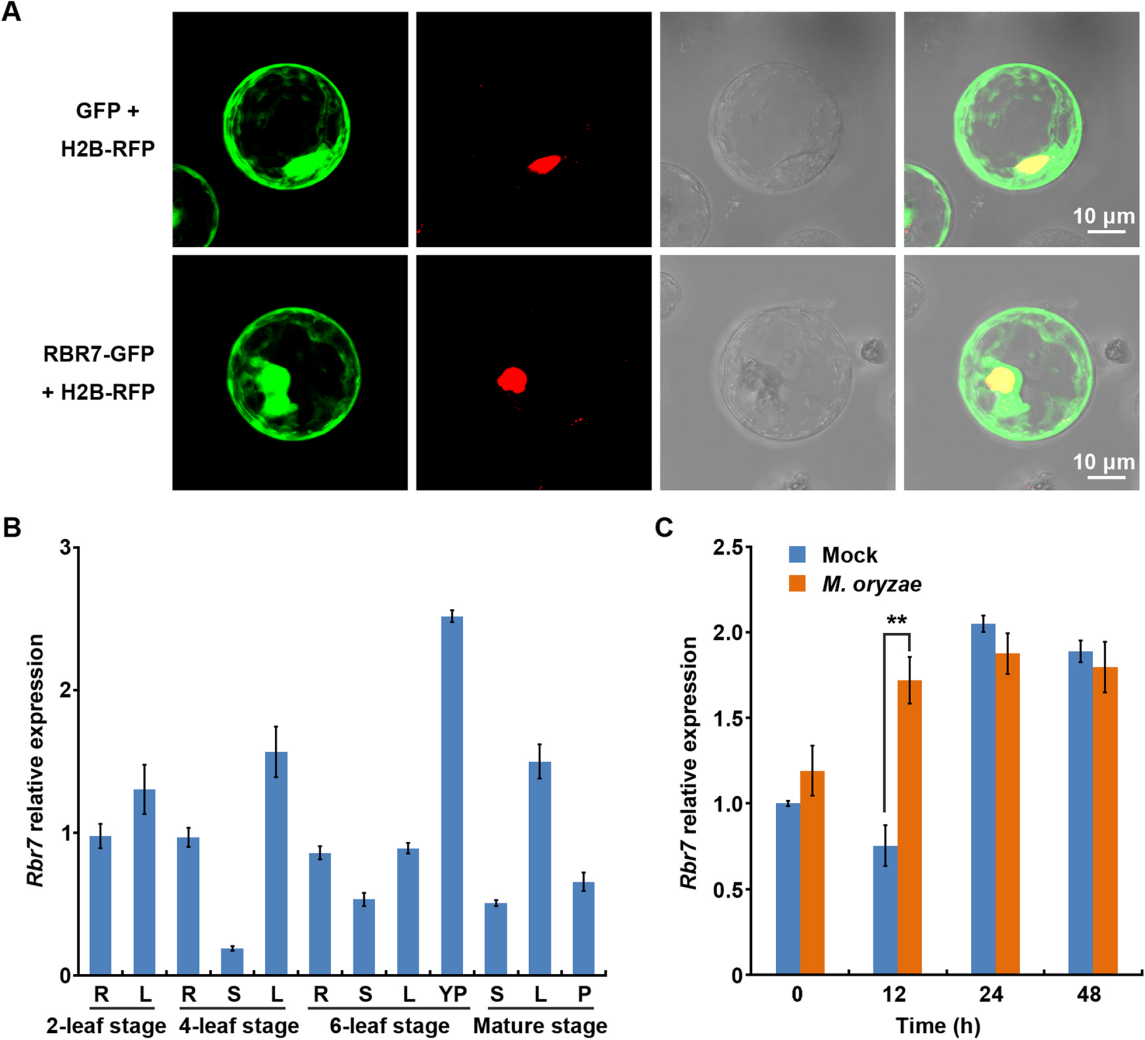
Characterization of RBR7. **A** Protein subcellular localization of RBR7 in rice protoplasts. As revealed from the merged green fluorescence of RBR7-GFP fusion protein and the red fluorescence of H2B-RFP (nuclear marker), RBR7 is localized in the nucleus and cytoplasm. Bars = 10 μm. **B** The expression level of *Rbr7* in different tissues of Kit plants from vegetative to reproductive stages. R, roots. L, leaf. S, stem. YP, young panicle. P, panicle. **C** The expression level of *Rbr7* post *M. oryzae* inoculation detected by qPCR. The values are means ± SD of three biological replicates per sample

Sequence analysis revealed that *Rbr7* encodes an unknown small protein with 85 amino acids. To characterize the function of RBR7, we examined the cellular localization of RBR7. We performed a rice protoplast transient assay with the pRTVc-*Rbr7*-GFP construct, in which the *Rbr7* coding sequence was fused in-frame with GFP to generate an RBR7-GFP fusion protein. The green fluorescence signals of the RBR7-GFP fusion protein were detected in the cytoplasm and nucleus of rice cells (Fig. 5A, Additional file 1: Fig. S8). This result indicated that the RBR7 protein may function in both the cytoplasm and the nucleus.

The expression pattern of *Rbr7* was analysed in different tissues of Kit plants from the vegetative to reproductive stages. The expression level of *Rbr7* was constitutive in different stages, suggesting that RBR7 functions throughout the entire rice growth period (Fig. 5B). Because of the genetic function of *Rbr7*, we tested whether the expression of *Rbr7* responds to pathogen infection*.* When inoculated with *M. oryzae* isolate Zhong10-8-14, the expression level of *Rbr7* was significantly induced in 12 h (Fig. 5C), suggesting that RBR7 plays a role at the early stage of the rice immune response.

### Upregulation of SA and Pathogenesis-Related Genes in *rbr7*

**Fig. 6:**
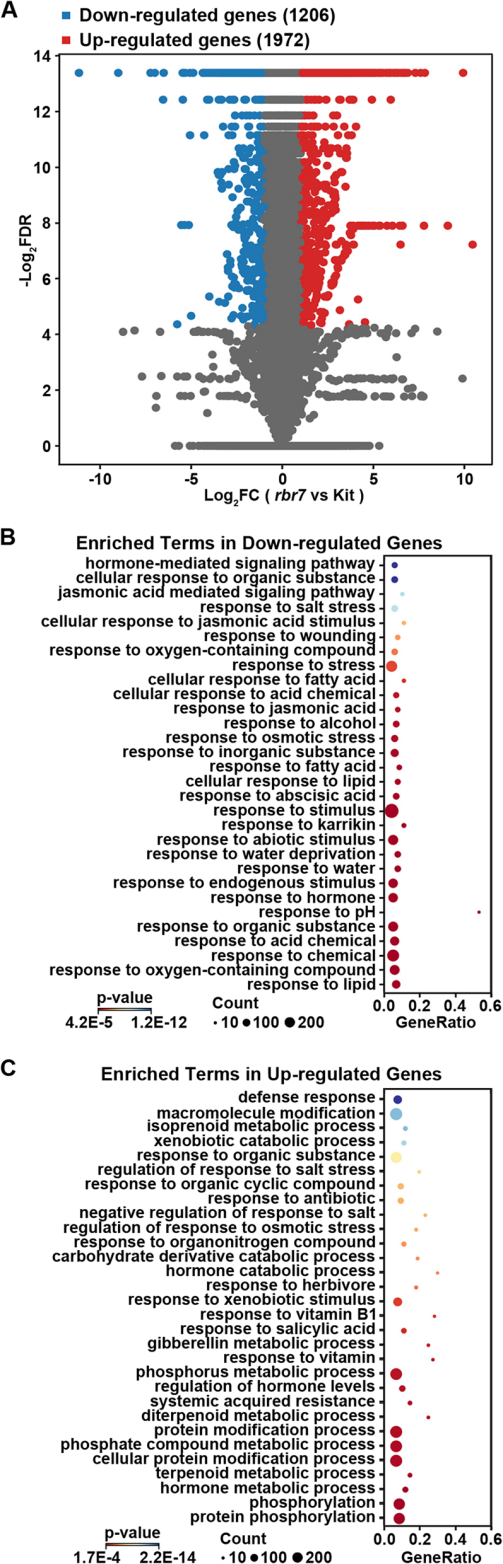
Transcriptomic analysis of *rbr7*. **A** Volcano plot of differentially expressed genes (DEGs) in *rbr7* compared with Kit. The DEGs were defined as FC (fold change) ≥ 2, FDR (false discovery rate) ≤ 0.05. **B** GO enrichment terms for the 1206 down-regulated genes in *rbr7* compared with Kit. **C** GO enrichment terms for the 1972 up-regulated genes in *rbr7* compared with Kit

**Fig. 7:**
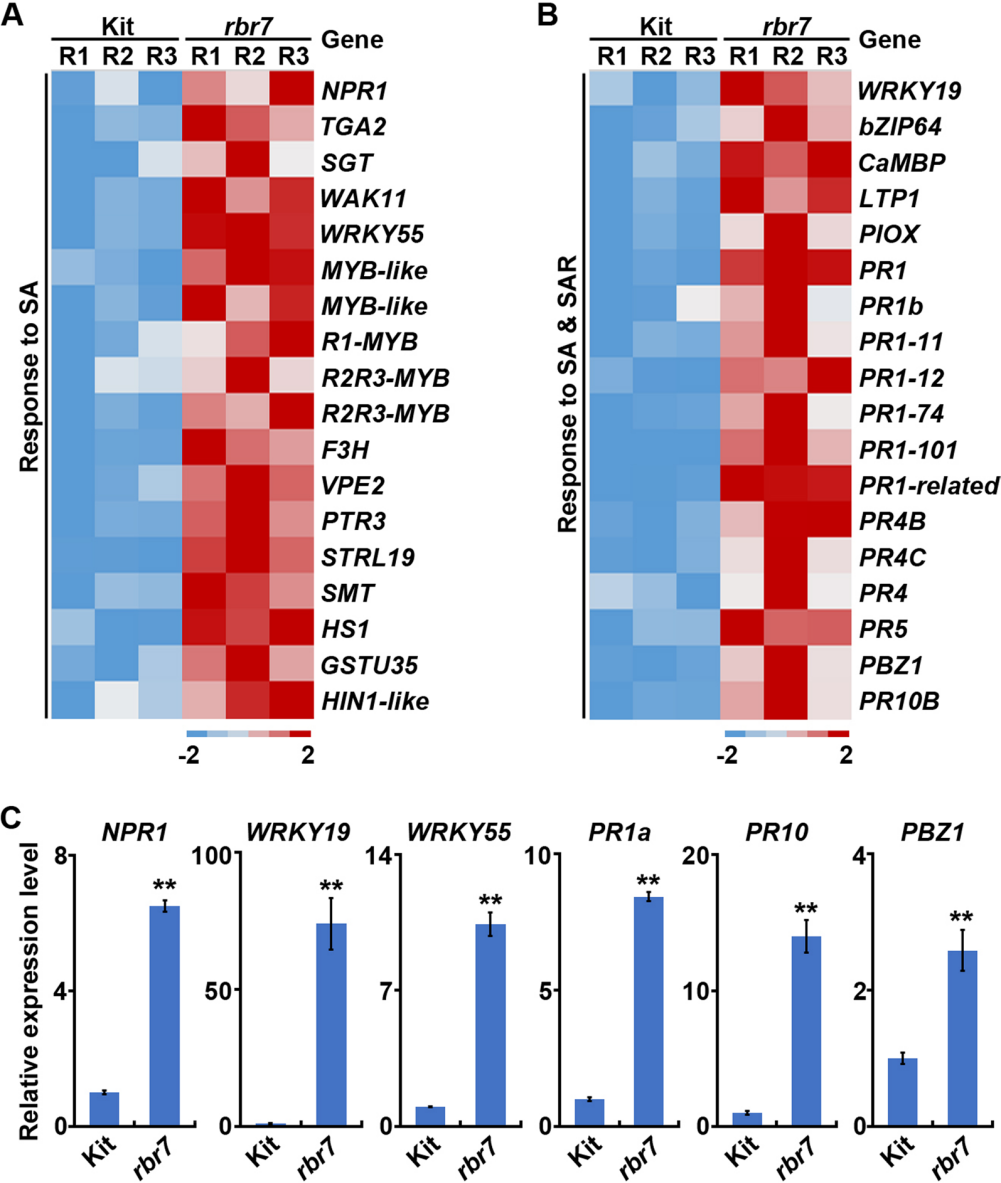
The responses to SA and SAR are changed in *rbr7*. **A** Heat map of the expression of genes under the GO term ‘Response to SA’ in *rbr7* compared with Kit. R1, R2 and R3 indicated three repeats for Kit and *rbr7* respectively. **B** Heat map of the expression of genes both under the GO term ‘Response to SA’ and ‘SAR’ in *rbr7* compared with Kit. R1, R2 and R3 indicated three repeats for Kit and *rbr7* respectively. **C** Expression levels of representative genes involved in the response to SA and SAR, including *NPR1*, *WRKY19*, *WRKY55*, *PR1a*, *PR10* and *PBZ1*. The values are means ± SD of three biological replicates per sample

To explore the molecular pathways regulated by RBR7, three-week-old leaves of Kit and the *rbr7* mutant were harvested for RNA-sequencing analysis. At this developmental stage, there were no visible mimic lesion spots on *rbr7*. A total of 3178 differentially expressed genes (DEGs) between Kit and the *rbr7* mutant were detected, of which 1972 genes were upregulated and 1206 genes were downregulated (Fig. 6A). The DEGs were analysed through enrichment analysis for Gene Ontology (GO) terms. Significantly enriched categories for downregulated genes were mainly related to response to inorganic substances, chemicals, lipids, stimuli, abiotic stimuli, and hormones (Fig. 6B). For the upregulated DEGs, the significantly enriched terms mainly involved the defence response, response to SA and SAR, relating to the activated immune response in the *rbr7* mutant (Fig. 6C). Furthermore, we found that the genes upregulated under the term ‘systemic acquired resistance’ were also included under the term ‘response to salicylic acid’ (Fig. 7A, B). Upregulation of the key immune component *NPR1* and transcription factor *TGA2* suggested activation of the SA signalling pathway. *SGT* encodes a key mediator of chemically induced blast resistance, which might contribute to the elevated resistance of the mutant *rbr7*. The induction of the transcription factors *WRKY19*, *WRKY55*, *bZIP64* and *MYB* family genes implied a wide range of activation of downstream immune response genes. *CaMBP* (*calmodulin binding protein*), *PIOX* (*pathogen-inducible oxygenase*), *VPE2* (*vacuolar processing enzyme 2*), *GSTU35* (*glutathione transferase U35*) and F3H (*flavanone 3-hydroxylase*) suggested protein and enzyme changes in the immunity regulatory networks. Furthermore, a series of pathogenesis-related (PR) genes, including *PR1*, *PR1b*, *PR1-11*, *PR1-12*, *PR4B*, *PR4C*, *PBZ1*, and *PR10B*, were also significantly upregulated, which might be closely related to defence activation and spontaneous cell death (Fig. 7A, B).

To further confirm this result, we analysed the expression levels of several representative genes in Kit and the *rbr7* mutant involved in the SA-mediated SAR signalling pathway by qRT‒PCR, including *NPR1* (*LOC_Os01g09800*), *WRKY19* (*LOC_Os05g49620*), *WRKY55* (*LOC_Os03g20550*), *PR1a* (*LOC_Os07g03710*), *PR10* (*LOC_Os12g36830*) and *PBZ1* (*LOC_Os12g36880*). All these genes showed significantly elevated expression in the *rbr7* mutant compared to Kit (Fig. 7C). Taken together, these results indicate activation of the SA signalling pathway in the *rbr7* mutant, leading to the elevated expression of various downstream genes regulating SAR and HR and enhanced immunity.

## Discussion

### RBR7 Negatively Regulates Rice Resistance to *M. oryzae*

Rice mutants with altered blast resistance are valuable resources to decipher the underlying mechanisms of the plant immune response. In this study, we identified a rice blast-resistant mutant *rbr7* from an EMS mutagenesis library of *japonica* rice (*Oryza sativa* cv. Kitaake). After inoculation with different isolates of *M. oryzae*, the *rbr7* mutant showed enhanced resistance compared with the wild-type Kit (Fig. 1, Additional file 1: Fig. S2). The *rbr7* mutant exhibited a typical light-induced lesion mimic phenotype with elevated ROS accumulation accompanied by cell death (Fig. 2). Through transcriptomic analysis, more genes were significantly upregulated in the *rbr7* mutant than in Kit (Fig. 6A). Furthermore, we found a significant induction of the genes, including *NPR1*, *TGA2*, *WRKY19*, *WRKY55* and a series of PR genes, falling to the category of response to SA and SAR (Fig. 7A, B), which was closely correlated to the spontaneous necrotic spot formation on the leaves of *rbr7* and the resistance phenotype of *rbr7*. These results imply that innate immunity was constitutively activated in *rbr7*, especially through upregulation of the SA signalling pathway, leading to elevated ROS levels, programmed cell death, SAR and enhanced resistance to *M. oryzae*.

### Spontaneous Lesion Formation in *rbr7* is Multifactor-Triggered

Previous studies have shown that spontaneous lesion appearance time, colour and shape can vary (Kang et al. 2021; Yin et al. 2000). Some lesion-mimicking spot formation is environmentally dependent (Sathe et al. 2019; Wang et al. 2015a, 2016; Yamanouchi et al. 2002). The *ell1* mutant is defective in chloroplast development and photosynthetic protein activation. Mimic lesion formation in *ell1* was light dependent and temperature induced (Cui et al. 2021). The light-shading experiment indicated that the spontaneous lesions on *rbr7* were light-dependent (Fig. 2B). Furthermore, we found that when *rbr7* plants were cultured in the growth chamber, lesion-mimicking spots did not form on the leaves (Additional file 1: Fig. S9). When *rbr7* plants were cultured in natural fields, spontaneous lesions appeared on the leaves, especially in environments where the temperature varied significantly. Therefore, we suggest that lesion-mimicking spot formation on *rbr7* is affected by environmental factors such as light and temperature.

Three-week-old *rbr7* plants, with no visible lesion mimic spots, showed enhanced resistance to rice blast, ROS accumulation, and cell death acceleration, suggesting that the activation of the immune response occurs prior to the initiation of leaf lesion mimic spots in *rbr7*. The *rbr7* mutant exhibited reduced plant height, its complemented plant had not restored its height to wild-type level (Additional file 1: Fig. S6), suggesting there might be other factors regulating its growth phenotype. Despite the uncertainty of the specific mechanisms mediated by RBR7, the role of RBR7 in regulating the immune response is demonstrated, and lesion-mimicking spot formation in *rbr7* is the result of a combination of overactivated immunity and environmental factors.

### *Rbr7* Encodes a Putative Uncharacterized Small Protein

*Rbr7* encodes a putative uncharacterized protein with 85 amino acids that has no predicted signature or domain. A search from the rice EST database in the Rice Genome Annotation Project (RGAP) showed evidence of the *Rbr7* transcript. Nevertheless, the translation of *Rbr7* is difficult to validate. Across species, small proteins of < 50 amino acids in prokaryotes or < 100 amino acids in eukaryotes are often missed in genome annotation and poorly characterized, as most small proteins are hard to identify with standard mass spectrometry (Gray et al. 2022; Steinberg and Koch 2021; Su et al. 2013). There are several categories of identified functional small proteins, including small secreted proteins, subunits of complexes, chaperones and other small open reading frames. Small secreted proteins are secreted by cells as defence regulators against pathogens or other stimuli (Hu et al. 2021). In plants, microproteins miP1a and miP1b were found to interact with CONSTANS and TOPLESS in a complex to regulate flowering time (Graeff et al. 2016). Small heat shock proteins and DnaJ proteins usually function as molecular chaperones or cochaperones regulating various cellular pathways (Chen et al. 2010; Waters and Vierling 2020). Other small open reading frames (smORFs) are in ‘untranslated regions’ or known as ‘noncoding’ RNAs (Malekos and Carpenter 2022).

BLAST analysis of the genome showed no homologue of RBR7 in rice. No homologues could be found in humans, mice, zebrafish, *Drosophila* or yeast. Homologues in other plants either have low homology with RBR7 in rice or are not characterized, which limits the characterization of RBR7. We speculate that RBR7 might work as a chaperone or a regulator within complexes. Its small size makes its translation faster and facilitates its diffusion or transport in cells and its reaction upon stimulus, contributing as a repressor or an enhancer towards different regulatory networks. Subcellular localization analysis indicated that RBR7 functions in the nucleus and cytoplasm, implying that it might work with transcription factors and/or other kinds of proteins. Transcriptomic analysis of Kit and *rbr7* suggested the possible regulatory role of RBR7 in rice, which might play a role as an enhancer of abiotic stimuli and a suppressor of biotic stress. The components involved in RBR7-mediated signalling pathway remains to be investigated, which is necessary to unravel the molecular mechanisms of RBR7 functioning in plant immunity or other development processes.

## Conclusions

Employing resistance genes in rice breeding is an effective strategy to protect plants from the threat of pathogens. In this research, we identified a rice mutant, *rbr7*, conferring enhanced resistance against different strains of *M. oryzae*, whose mutation resulted from the disruption of *Rbr7*, encoding the previously uncharacterized small protein RBR7. The disruption of RBR7 in rice leads to activation of the SA signalling pathway, upregulation of PR genes and ROS accumulation, ultimately leading to programmed cell death and enhanced disease resistance. These results shed light on novel small proteins that play pivotal roles in the rice immune response and provide valuable resources for rice breeding. However, the function of RBR7 remains largely unknown due to the limited available information about this protein. A possible strategy is to determine whether the complex RBR7 might function in or interact with a protein that serves in the rice immune response or other processes.

### Supplementary Information

**Additional file 1.** **Fig. S1.** Leaf phenotype of *rbr7* before M.oryzae inoculation. **Fig. S2.** Rice blast fungus resistance phenotype of *rbr7* with punch inoculation. **Fig. S3.** Cosegregation analysis of the deletion region inr *br7*. **Fig. S4.** Expression of adjacent genes to the deletion region in *rbr7*. **Fig. S5.** Rice blast inoculation phenotype of the complemented lines. **Fig. S6.** Morphological phenotype of Kit, *rbr7* and *Rbr7-comp*. **Fig. S7.** Rice blast inoculation phenotype of the knockout lines. **Fig. S8.** Detection of RBR7-GFP protein in subcellular localization. **Fig. S9.** Leaf phenotype of *rbr7* in a growth chamber.

**Additional file 2. Table S1.** Genetic analysis of the *rbr7* mutant phenotype.

**Additional file 3. Table S2.** Primers used in this research.

**Additional file 4.** Genes in the 400-kb candidate region.

**Additional file 5.** Mutated allele frequency of *rbr7* and Kit phenotype pool from F2 population in the candidate region.

**Additional file 6.** Gene expression of 3178 DEGs between Kit and the rbr7 mutant from the transcriptomic sequencing.

## Data Availability

The BSA sequencing data and the RNA-sequencing data could be retrieved with the accession number PRJCA019370 and PRJCA016897 at the National Genomics Data Center (https://ngdc.cncb.ac.cn), respectively.

## Abbreviations

*rbr7*: Rice blast resistance 7
ROS: Reactive oxygen species
SA: Salicylic acid
*M. oryzae*: *Magnaporthe oryzae*
SAR: Systemic acquired resistance
HR: Hypersensitive response
DAB: 3,3′-Diaminobenzidine
6-BA: 6-Benzylaminopurine
UBQ: Ubiquitin
InDel: Insertion–deletion
SSR: Simple sequence repeat
BSA: Bulk segregation analysis
EMS: Ethyl methane sulfonate
Kit: Kitaake
DEGs: Differentially expressed genes
GO: Gene ontology
smORFs: Small open reading frames

## References

[CR1] Bart R, Chern M, Park CJ, Bartley L, Ronald PC (2006). A novel system for gene silencing using siRNAs in rice leaf and stem-derived protoplasts. Plant Methods.

[CR2] Cao H, Bowling SA, Gordon AS, Dong X (1994). Characterization of an *Arabidopsis* mutant that is nonresponsive to inducers of systemic acquired resistance. Plant Cell.

[CR3] Cao H, Glazebrook J, Clarke JD, Volko S, Dong X (1997). The Arabidopsis *NPR1* gene that controls systemic acquired resistance encodes a novel protein containing ankyrin repeats. Cell.

[CR4] Chen KM, Holmstrom M, Raksajit W, Suorsa M, Piippo M, Aro EM (2010). Small chloroplast-targeted DnaJ proteins are involved in optimization of photosynthetic reactions in *Arabidopsis thaliana*. BMC Plant Biol.

[CR5] Chen XF, Hao L, Pan JW, Zheng XX, Jiang GH, Jin Y, Gu ZM, Qian Q, Zhai WX, Ma BJ (2012). *SPL5*, a cell death and defense-related gene, encodes a putative splicing factor 3b subunit 3 (SF3b3) in rice. Mol Breed.

[CR6] Chern M, Canlas PE, Fitzgerald HA, Ronald PC (2005). Rice NRR, a negative regulator of disease resistance, interacts with Arabidopsis NPR1 and rice NH1. Plant J.

[CR7] Coll NS, Epple P, Dangl JL (2011). Programmed cell death in the plant immune system. Cell Death Differ.

[CR8] Cui Y, Peng Y, Zhang Q, Xia S, Ruan B, Xu Q, Yu X, Zhou T, Liu H, Zeng D, Zhang G, Gao Z, Hu J, Zhu L, Shen L, Guo L, Qian Q, Ren D (2021). Disruption of *EARLY LESION LEAF 1*, encoding a cytochrome P450 monooxygenase, induces ROS accumulation and cell death in rice. Plant J.

[CR9] Donahue JL, Alford SR, Torabinejad J, Kerwin RE, Nourbakhsh A, Ray WK, Hernick M, Huang XY, Lyons BM, Hein PP, Gillaspy GE (2010). The *Arabidopsis thaliana Myo-Inositol 1-Phosphate Synthase1* gene is required for myo-inositol synthesis and suppression of cell death. Plant Cell.

[CR10] Graeff M, Straub D, Eguen T, Dolde U, Rodrigues V, Brandt R, Wenkel S (2016). MicroProtein-mediated recruitment of CONSTANS into a TOPLESS trimeric complex represses flowering in *Arabidopsis*. PLoS Genet.

[CR11] Gray J, Janick-Buckner D, Buckner B, Close PS, Johal GS (2002). Light-dependent death of maize *lls1* cells is mediated by mature chloroplasts. Plant Physiol.

[CR12] Gray T, Storz G, Papenfort K (2022). Small proteins; Big Questions. J Bacteriol.

[CR13] Greenberg JT, Silverman FP, Liang H (2000). Uncoupling salicylic acid-dependent cell death and defense-related responses from disease resistance in the Arabidopsis mutant *acd5*. Genetics.

[CR14] Hu G, Yalpani N, Briggs SP, Johal GS (1998). A porphyrin pathway impairment is responsible for the phenotype of a dominant disease lesion mimic mutant of maize. Plant Cell.

[CR15] Hu XL, Lu H, Hassan MM, Zhang J, Yuan G, Abraham PE, Shrestha HK, Villalobos Solis MI, Chen JG, Tschaplinski TJ, Doktycz MJ, Tuskan GA, Cheng ZM, Yang X (2021). Advances and perspectives in discovery and functional analysis of small secreted proteins in plants. Hortic Res.

[CR16] Jabs T, Dietrich RA, Dangl JL (1996). Initiation of runaway cell death in an *Arabidopsis* mutant by extracellular superoxide. Science.

[CR17] Jones JD, Dangl JL (2006). The plant immune system. Nature.

[CR18] Kang SG, Lee KE, Singh M, Kumar P, Matin MN (2021). Rice lesion mimic mutants (LMM): the current understanding of genetic mutations in the failure of ROS scavenging during lesion formation. Plants (basel).

[CR19] Kumar S, Zavaliev R, Wu Q, Zhou Y, Cheng J, Dillard L, Powers J, Withers J, Zhao J, Guan Z, Borgnia MJ, Bartesaghi A, Dong X, Zhou P (2022). Structural basis of NPR1 in activating plant immunity. Nature.

[CR20] Liu R, Lu J, Zheng S, Du M, Zhang C, Wang M, Li Y, Xing J, Wu Y, Zhang L (2021). Molecular mapping of a novel lesion mimic gene (*lm4*) associated with enhanced resistance to stripe rust in bread wheat. BMC Genom Data.

[CR21] Malamy J, Carr JP, Klessig DF, Raskin I (1990). Salicylic Acid: a likely endogenous signal in the resistance response of tobacco to viral infection. Science.

[CR22] Malekos E, Carpenter S (2022). Short open reading frame genes in innate immunity: from discovery to characterization. Trends Immunol.

[CR23] Metraux JP, Signer H, Ryals J, Ward E, Wyss-Benz M, Gaudin J, Raschdorf K, Schmid E, Blum W, Inverardi B (1990). Increase in salicylic acid at the onset of systemic acquired resistance in cucumber. Science.

[CR24] Mizobuchi R, Hirabayashi H, Kaji R, Nishizawa Y, Yoshimura A, Satoh H, Ogawa T, Okamoto M (2002). Isolation and characterization of rice lesion-mimic mutants with enhanced resistance to rice blast and bacterial blight. Plant Sci.

[CR25] Morel JB, Dangl JL (1997). The hypersensitive response and the induction of cell death in plants. Cell Death Differ.

[CR26] Mori M, Tomita C, Sugimoto K, Hasegawa M, Hayashi N, Dubouzet JG, Ochiai H, Sekimoto H, Hirochika H, Kikuchi S (2007). Isolation and molecular characterization of a *Spotted leaf 18* mutant by modified activation-tagging in rice. Plant Mol Biol.

[CR27] Morris SW, Vernooij B, Titatarn S, Starrett M, Thomas S, Wiltse CC, Frederiksen RA, Bhandhufalck A, Hulbert S, Uknes S (1998). Induced resistance responses in maize. Mol Plant Microbe Interact.

[CR28] Mou Z, He Y, Dai Y, Liu X, Li J (2000). Deficiency in fatty acid synthase leads to premature cell death and dramatic alterations in plant morphology. Plant Cell.

[CR29] Mur LA, Kenton P, Lloyd AJ, Ougham H, Prats E (2007). The hypersensitive response; the centenary is upon us but how much do we know?. J Exp Bot.

[CR30] Ngou BPM, Ahn HK, Ding P, Jones JDG (2021). Mutual potentiation of plant immunity by cell-surface and intracellular receptors. Nature.

[CR31] Noutoshi Y, Kuromori T, Wada T, Hirayama T, Kamiya A, Imura Y, Yasuda M, Nakashita H, Shirasu K, Shinozaki K (2006). Loss of *Necrotic Spotted Lesions 1* associates with cell death and defense responses in *Arabidopsis thaliana*. Plant Mol Biol.

[CR32] Pruitt RN, Locci F, Wanke F, Zhang L, Saile SC, Joe A, Karelina D, Hua C, Frohlich K, Wan WL, Hu M, Rao S, Stolze SC, Harzen A, Gust AA, Harter K, Mhaj J, Bphj T, Zhou JM, Dangl JL, Weigel D, Nakagami H, Oecking C, Kasmi FE, Parker JE, Nurnberger T (2021). The EDS1-PAD4-ADR1 node mediates *Arabidopsis* pattern-triggered immunity. Nature.

[CR33] Qiao Y, Jiang W, Lee J, Park B, Choi MS, Piao R, Woo MO, Roh JH, Han L, Paek NC, Seo HS, Koh HJ (2010). *SPL28* encodes a clathrin-associated adaptor protein complex 1, medium subunit μ1 (AP1M1) and is responsible for spotted leaf and early senescence in rice (*Oryza sativa*). New Phytol.

[CR34] Qiu TC, Zhao XS, Feng HJ, Qi LL, Yang J, Peng YL, Zhao WS (2021). OsNBL3, a mitochondrion-localized pentatricopeptide repeat protein, is involved in splicing *nad5* intron 4 and its disruption causes lesion mimic phenotype with enhanced resistance to biotic and abiotic stresses. Plant Biotechnol J.

[CR35] Sakamoto K, Tada Y, Yokozeki Y, Akagi H, Hayashi N, Fujimura T, Ichikawa N (1999). Chemical induction of disease resistance in rice is correlated with the expression of a gene encoding a nucleotide binding site and leucine-rich repeats. Plant Mol Biol.

[CR36] Sathe AP, Su X, Chen Z, Chen T, Wei X, Tang S, Zhang XB, Wu JL (2019). Identification and characterization of a spotted-leaf mutant *spl40* with enhanced bacterial blight resistance in rice. Rice (N Y).

[CR37] Silverman P, Seskar M, Kanter D, Schweizer P, Metraux JP, Raskin I (1995). Salicylic acid in rice (Biosynthesis, conjugation, and possible role). Plant Physiol.

[CR38] Steinberg R, Koch HG (2021). The largely unexplored biology of small proteins in pro- and eukaryotes. FEBS J.

[CR39] Su M, Ling Y, Yu J, Wu J, Xiao J (2013). Small proteins: untapped area of potential biological importance. Front Genet.

[CR40] Tang J, Zhu X, Wang Y, Liu L, Xu B, Li F, Fang J, Chu C (2011). Semi-dominant mutations in the CC-NB-LRR-type R gene, *NLS1*, lead to constitutive activation of defense responses in rice. Plant J.

[CR41] Thordal-Christensen H, Zhang Z, Wei Y, Collinge DB (1997). Subcellular localization of H_2_O_2_ in plants. H_2_O_2_ accumulation in papillae and hypersensitive response during the barley—powdery mildew interaction. Plant J.

[CR42] Tsuda K, Sato M, Stoddard T, Glazebrook J, Katagiri F (2009). Network properties of robust immunity in plants. PLoS Genet.

[CR43] Vlot AC, Dempsey DA, Klessig DF (2009). Salicylic Acid, a multifaceted hormone to combat disease. Annu Rev Phytopathol.

[CR44] Wang J, Ye B, Yin J, Yuan C, Zhou X, Li W, He M, Wang J, Chen W, Qin P, Ma B, Wang Y, Li S, Chen X (2015). Characterization and fine mapping of a light-dependent *leaf lesion mimic mutant 1* in rice. Plant Physiol Biochem.

[CR45] Wang SH, Lim JH, Kim SS, Cho SH, Yoo SC, Koh HJ, Sakuraba Y, Paek NC (2015). Mutation of SPOTTED LEAF3 (SPL3) impairs abscisic acid-responsive signalling and delays leaf senescence in rice. J Exp Bot.

[CR46] Wang F, Wu W, Wang D, Yang W, Sun J, Liu D, Zhang A (2016). Characterization and genetic analysis of a novel light-dependent lesion mimic mutant, *lm3*, showing adult-plant resistance to powdery mildew in common wheat. PLoS ONE.

[CR47] Wang J, Shi H, Zhou L, Peng CF, Liu DY, Zhou XG, Wu WG, Yin JJ, Qin H, Ma WW, He M, Li WT, Wang JC, Li SG, Chen XW (2017). OsBSK1-2, an orthologous of AtBSK1, is involved in rice immunity. Front Plant Sci.

[CR48] Wang J, Yu H, Xiong GS, Lu ZF, Jiao YQ, Meng XB, Liu GF, Chen XW, Wang YH, Li JY (2017). Tissue-specific ubiquitination by IPA1 INTERACTING PROTEIN1 modulates IPA1 protein levels to regulate plant architecture in rice. Plant Cell.

[CR49] Wang S, Lei C, Wang J, Ma J, Tang S, Wang C, Zhao K, Tian P, Zhang H, Qi C, Cheng Z, Zhang X, Guo X, Liu L, Wu C, Wan J (2017). *SPL33*, encoding an eEF1A-like protein, negatively regulates cell death and defense responses in rice. J Exp Bot.

[CR50] Waters ER, Vierling E (2020). Plant small heat shock proteins—evolutionary and functional diversity. New Phytol.

[CR51] Wu C, Bordeos A, Madamba MR, Baraoidan M, Ramos M, Wang GL, Leach JE, Leung H (2008). Rice lesion mimic mutants with enhanced resistance to diseases. Mol Genet Genomics.

[CR52] Xiao GQ, Zhou JH, Lu XY, Huang RF, Zhang HW (2018). Excessive UDPG resulting from the mutation of *UAP1* causes programmed cell death by triggering reactive oxygen species accumulation and caspase-like activity in rice. New Phytol.

[CR53] Yamanouchi U, Yano M, Lin H, Ashikari M, Yamada K (2002). A rice spotted leaf gene, *Spl7*, encodes a heat stress transcription factor protein. Proc Natl Acad Sci U S A.

[CR54] Yang Y, Qi M, Mei C (2004). Endogenous salicylic acid protects rice plants from oxidative damage caused by aging as well as biotic and abiotic stress. Plant J.

[CR55] Yang L, Li BS, Zheng XY, Li JG, Yang M, Dong XN, He GM, An CC, Deng XW (2015). Salicylic acid biosynthesis is enhanced and contributes to increased biotrophic pathogen resistance in *Arabidopsis* hybrids. Nat Commun.

[CR56] Yao Q, Zhou R, Fu T, Wu W, Zhu Z, Li A, Jia J (2009). Characterization and mapping of complementary lesion-mimic genes *lm1* and *lm2* in common wheat. Theor Appl Genet.

[CR57] Yin Z, Chen J, Zeng L, Goh M, Leung H, Khush GS, Wang GL (2000). Characterizing rice lesion mimic mutants and identifying a mutant with broad-spectrum resistance to rice blast and bacterial blight. Mol Plant Microbe Interact.

[CR58] Yuan M, Jiang Z, Bi G, Nomura K, Liu M, Wang Y, Cai B, Zhou JM, He SY, Xin XF (2021). Pattern-recognition receptors are required for NLR-mediated plant immunity. Nature.

[CR59] Zavaliev R, Mohan R, Chen T, Dong X (2020). Formation of NPR1 condensates promotes cell survival during the plant immune response. Cell.

[CR60] Zeng LR, Qu S, Bordeos A, Yang C, Baraoidan M, Yan H, Xie Q, Nahm BH, Leung H, Wang GL (2004). *Spotted leaf11*, a negative regulator of plant cell death and defense, encodes a U-box/armadillo repeat protein endowed with E3 ubiquitin ligase activity. Plant Cell.

[CR61] Zhang A, Jiang H, Chu H, Cao L, Chen J (2022). Rice lesion mimic gene cloning and association analysis for disease resistance. Curr Issues Mol Biol.

[CR62] Zhu X, Yin J, Liang S, Liang R, Zhou X, Chen Z, Zhao W, Wang J, Li W, He M, Yuan C, Miyamoto K, Ma B, Wang J, Qin P, Chen W, Wang Y, Wang W, Wu X, Yamane H, Zhu L, Li S, Chen X (2016). The Multivesicular bodies (MVBs)-localized AAA ATPase LRD6-6 inhibits immunity and cell death likely through regulating MVBs-mediated vesicular trafficking in rice. PLoS Genet.

[CR63] Zhu XB, Ze M, Yin JJ, Chern M, Wang MR, Zhang X, Deng R, Li YZ, Liao HC, Wang L, Tu B, Song L, He M, Li SG, Wang WM, Chen XW, Wang J, Li WT (2020). A phosphofructokinase B-type carbohydrate kinase family protein, PFKB1, is essential for chloroplast development at early seedling stage in rice. Plant Sci.

[CR64] Zhu X, Ze M, Chern M, Chen X, Wang J (2020). Deciphering rice lesion mimic mutants to understand molecular network governing plant immunity and growth. Rice Sci.

