# Supplementary material for: Disruption of the Novel Small Protein RBR7 Leads to Enhanced Plant Resistance to Blast Disease
Source: Rice (N Y). 2023 Sep 21;16:42. doi: 10.1186/s12284-023-00660-1 (PMC10513991; doi:10.1186/s12284-023-00660-1)
Supplement: Supplementary file 1 — Additional file 1. Fig. S1. Leaf phenotype of rbr7 before M.oryzae inoculation. Fig. S2. Rice blast fungus resistance phenotype of rbr7 with punch inoculation. Fig. S3. Cosegregation analysis of the deletion region inr br7. Fig. S4. Expression of adjacent genes to the deletion region in rbr7. Fig. S5. Rice blast inoculation phenotype of the complemented lines. Fig. S6. Morphological phenotype of Kit, rbr7 and Rbr7-comp. Fig. S7. Rice blast inoculation phenotype of the knockout lines. Fig. S8. Detection of RBR7-GFP protein in subcellular localization. Fig. S9. Leaf phenotype of rbr7 in a growth chamber. [file 12284_2023_660_MOESM1_ESM.docx]

**
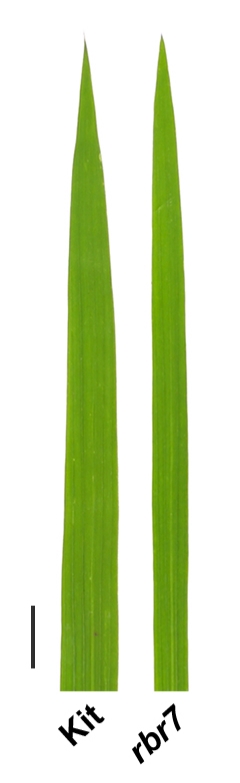
**

**Fig. S1. Leaf phenotype of *rbr7* before *M.oryzae* inoculation.** Leaves from Kit and *rbr7* grown in the field prior to mimic lesion formation on *rbr7* (From about three-week-old plants). Bar = 1 cm.


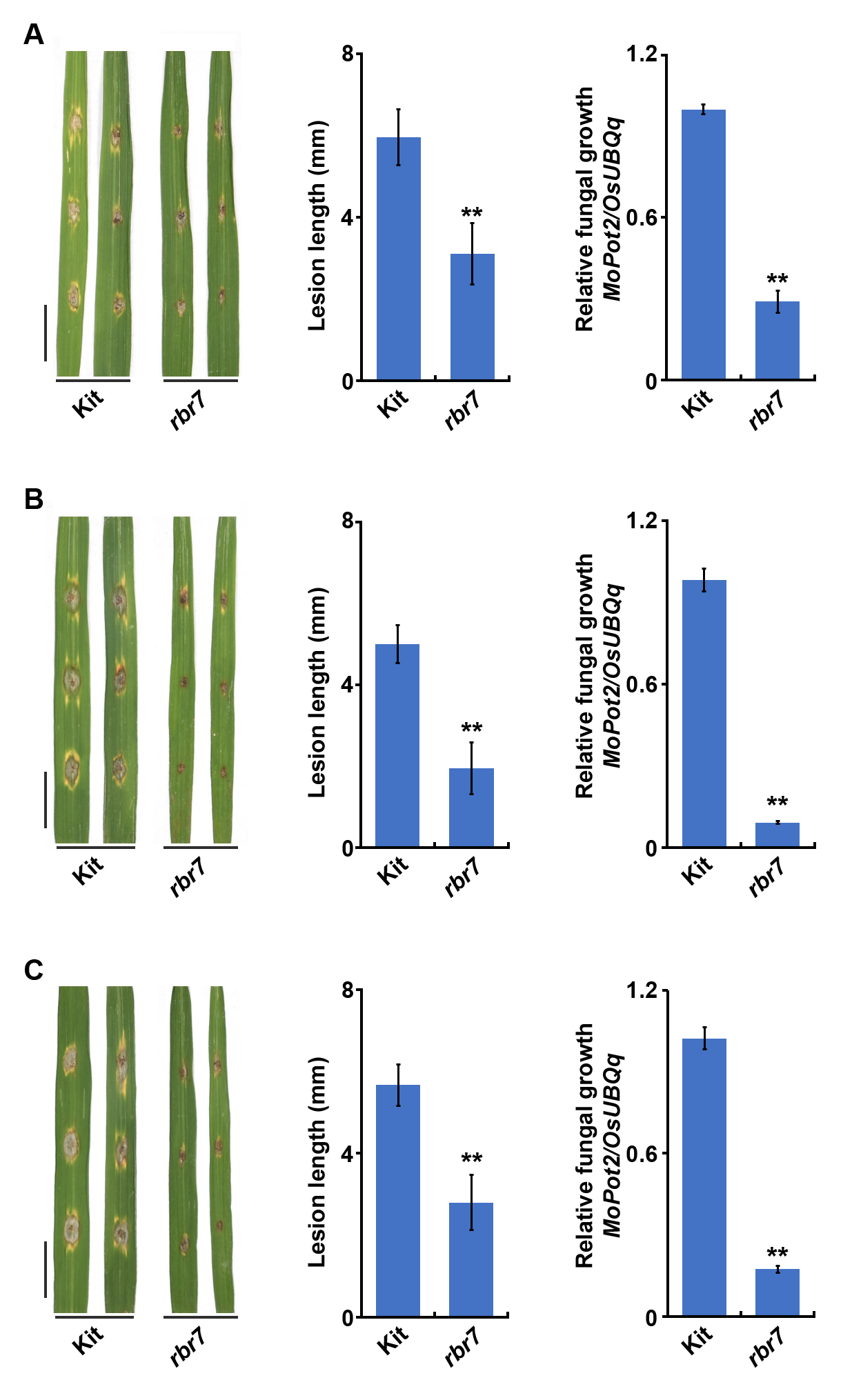


**Fig. S2. Rice blast fungus resistance phenotype of *rbr7* with punch inoculation.** A. Leaves of Kit and *rbr7* after punch inoculation with spores of *M. oryzae* isolates Zhong10-8-14, ZE-1 and 0755-1-1. Bars =1 cm. B. Lesion length on leaves represented in (A). The values are means ± SD of 10 lesions per sample. The asterisks indicate significant difference compared with Kit (P < 0.01, student’s *t* test). C. Relative fungal growth in inoculated leaves of Kit and *rbr7* detected by qPCR of *MoPot2* relative to *UBQ* gDNA level. The values are means ± SD of three biological replicates per sample.


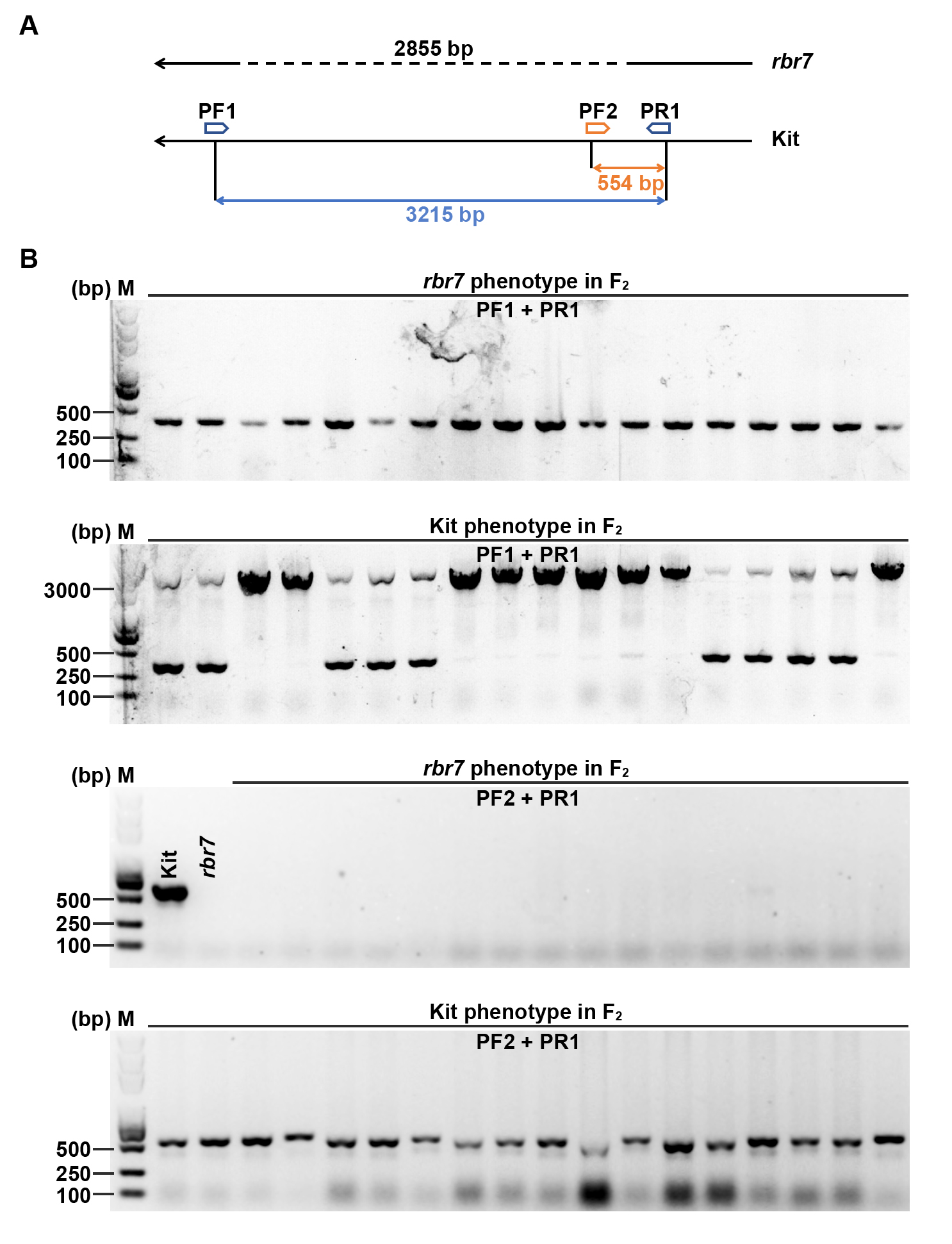


**Fig. S3. Cosegregation analysis of the deletion region in *rbr7***. A. A diagram of the deletion region in *rbr7* and the primers used in segregation analysis. The dashed line indicated the deletion region in *rbr7*. PF1, PF2, and PR1 indicated the primer locations in and around of the deletion region. The blue and red arrowheads indicate PF1 + PR1, PF2 + PR1 PCR product length respectively. B. PCR product lengths of *rbr7* phenotype individuals from F_2_ population and Kit phenotype individuals from F_2_ population with the primer pairs PF1 + PR1 and PF2 + PR1, respectively. M represents DNA marker (Tsingke DL5000 DNA marker).


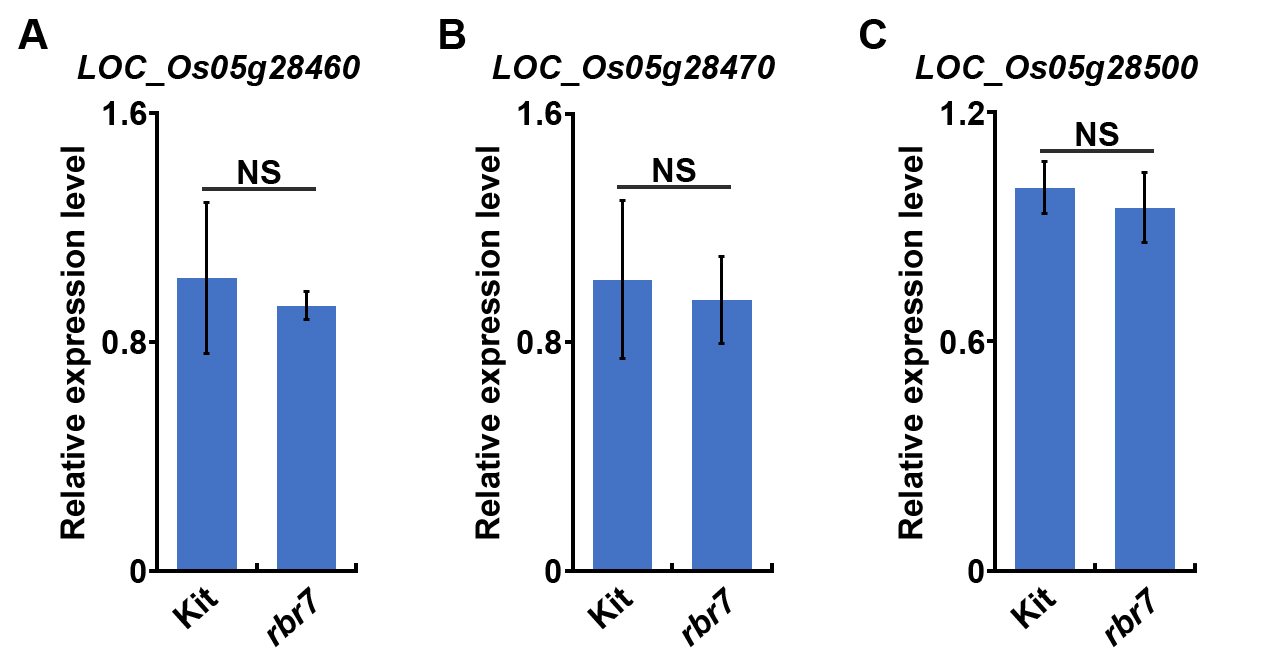


**Fig. S4.** **Expression of adjacent genes to the deletion region in *rbr7*.** A-C. Expression of *LOC_Os05g28460* (A), *LOC_Os05g28470* (B) and *LOC_Os05g28500* (C) which are adjacent to the deletion region in leaves of Kit and *rbr7* detected by qPCR relative to *UBQ* expression. The values are means ± SD of three biological replicates per sample.

**
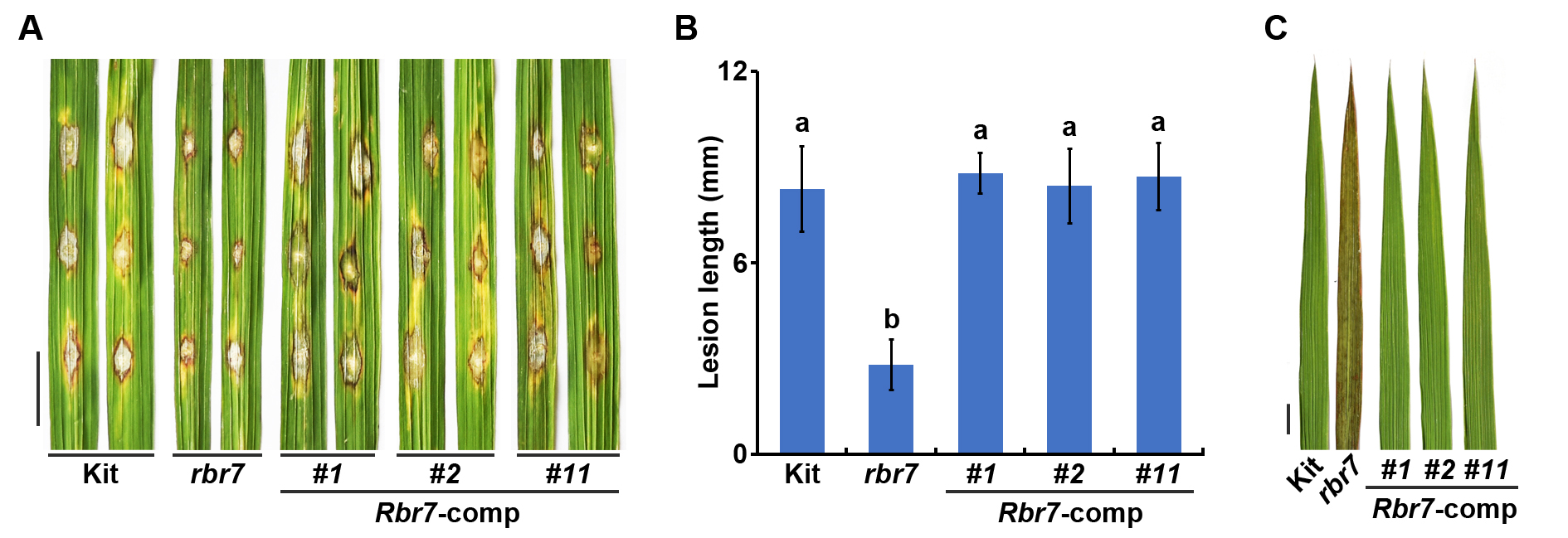
**

**Fig. S5.** **Rice blast inoculation phenotype of the complemented lines.** A. Leaves of Kit, *rbr7* and *Rbr7*-comp (complemented lines of *rbr7*) after punch inoculation with the *M. oryzae* isolate Zhong10-8-14. Three-week-old seedlings were used. Bar = 1 cm. B. Lesion length on leaves represented in (A). The values are means ± SD of 10 lesions per sample. Letters above each column indicate significant difference between the compared pairs (P < 0.05, one-way ANOVA with Tukey’s test). C. Leaves from Kit, *rbr7* and *Rbr7*-comp grown in the natural field. Bar = 1 cm.


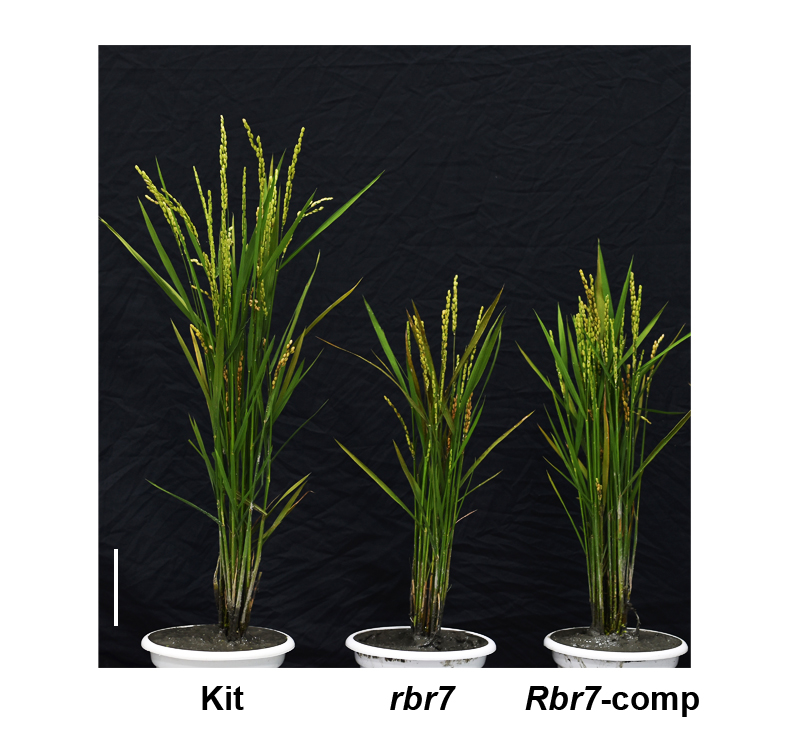


**Fig. S6. Morphological phenotype of Kit, *rbr7* and *Rbr7*-comp.** Representative plant of Kit, *rbr7* and *Rbr7*-comp grown in the field. Bar = 10 cm.


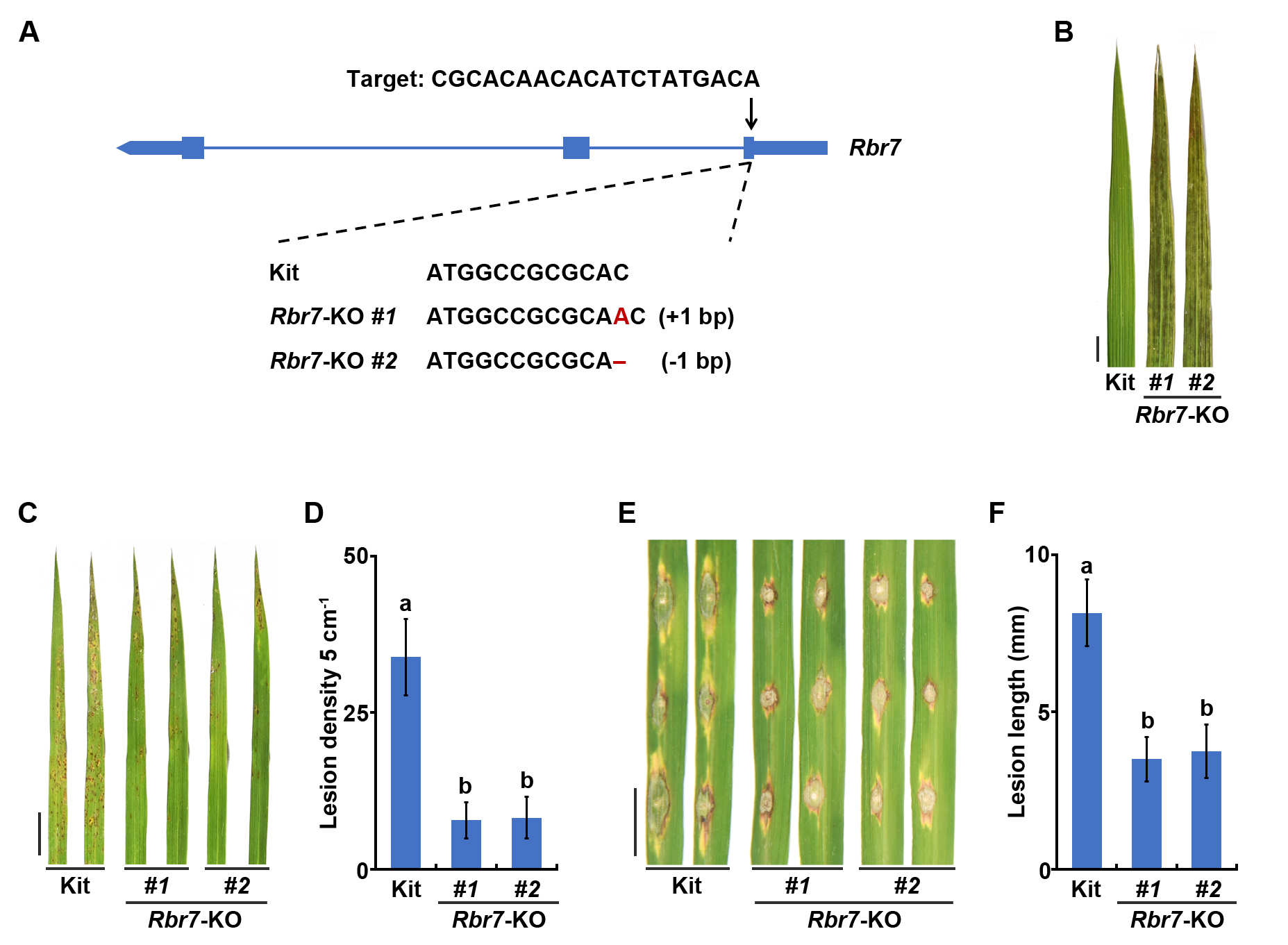


**Fig. S7. Rice blast inoculation phenotype of the knockout lines.** A. CRISPR/Cas9 target site and genotypes of *Rbr7* knockout lines. B. Leaves from Kit and *Rbr7* knockout lines (*Rbr7*-KO) grown in the natural field. Bar = 1 cm. C. Leaves of Kit, *Rbr7* knockout lines (*Rbr7*-KO) after spray inoculation with the *M. oryzae* isolate Zhong10-8-14. Three-week-old seedlings were used. Bar = 1 cm. D. Lesion density per 5 cm on leaves represented in (C). The values are means ± SD of 10 leaves per sample. Letters above each column indicate significant difference between the compared pairs (P < 0.05, one-way ANOVA with Tukey’s test). E. Leaves of Kit, *Rbr7* knockout lines (*Rbr7*-KO) after punch inoculation with the *M. oryzae* isolate Zhong10-8-14. Three-week-old seedlings were used. Bar = 1 cm. F. Lesion length on leaves represented in (E). The values are means ± SD of 10 lesions per sample. Letters above each column indicate significant difference between the compared pairs (P < 0.05, one-way ANOVA with Tukey’s test).


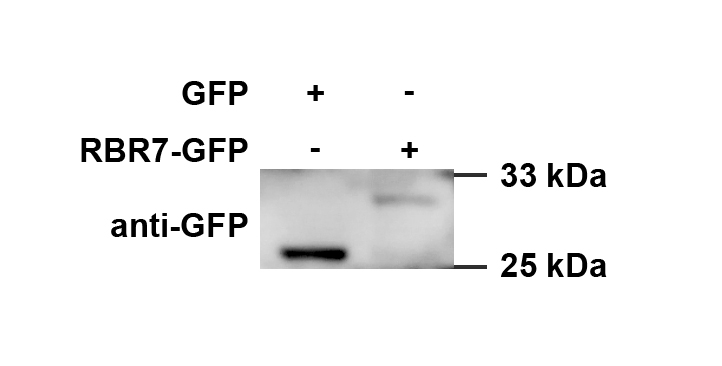


**Fig. S8.** **Detection of RBR7-GFP protein in subcellular localization.** Immunoblots of the GFP and RBR7-GFP protein from rice protoplast transient assay for subcellular localization.


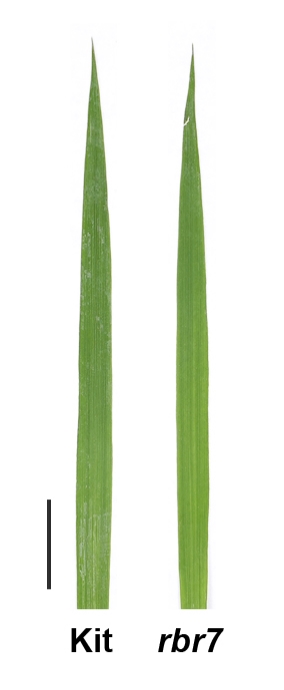


**Fig. S9.** **Leaf phenotype of *rbr7* in a growth chamber.** Four-week-old leaves from Kit and *rbr7* grown in a growth chamber. No mimic lesion was detected on the leaf of *rbr7*. Bar = 1 cm.
