# Supplementary material for: Disruption of the Novel Small Protein RBR7 Leads to Enhanced Plant Resistance to Blast Disease
Source: Rice (N Y). 2023 Sep 21;16:42. doi: 10.1186/s12284-023-00660-1 (PMC10513991; doi:10.1186/s12284-023-00660-1)
Supplement: Supplementary file 2 — Additional file 2. Table S1. Genetic analysis of the rbr7 mutant phenotype. [file 12284_2023_660_MOESM2_ESM.docx]

Table S1. Genetic analysis of the *rbr7* mutant phenotype.

| Cross | F1 population | F2 population | | χ^2^(3 : 1) |
| --- | --- | --- | --- | --- |
|  |  | Kit phenotype | Mutant phenotype |  |
| Kit × *rbr7* | Kit phenotype | 359 | 101 | 2.27 |
| *rbr7* × Kit | Kit phenotype | 215 | 88 | 2.64 |
| Jodan × *rbr7* | Kit phenotype | 1650 | 506 | 2.69 |

Note: Critical value (1, 0.05) = 3.84
