# Supplementary material for: Disruption of the Novel Small Protein RBR7 Leads to Enhanced Plant Resistance to Blast Disease
Source: Rice (N Y). 2023 Sep 21;16:42. doi: 10.1186/s12284-023-00660-1 (PMC10513991; doi:10.1186/s12284-023-00660-1)
Supplement: Supplementary file 3 — Additional file 3. Table S2. Primers used in this research. [file 12284_2023_660_MOESM3_ESM.docx]

Table S2. Primers used in this research

| Primer Name | Sequence (5’-3’) | |
| --- | --- | --- |
| Primers for mapping the *Rbr7* locus | | |
| RM18380-F | CGATCGTCCATTAGCTTTCTCG | |
| RM18380-R | TAAGGACCTAGCCGATGCAACC | |
| RM440-F | GGTAGGCACCAAAGAGTTTGACG | |
| RM440-R | GGCATCACCTTATCCAATCACC | |
| RM18522-F | ACCTCCTCGCTCGTCTCTCTCC | |
| RM18522-R | CCTGCCTCGTGAAGTTGAGAGC | |
| I21-F | AGCTGTACGAGGAGTGGA | |
| I21-R | GTAATCCGATTCTGTGGT | |
| I26-F | CTCCTACCCGCCACCACTA | |
| I26-R | GAGGTCCACCTGCGAAAA | |
| GM1-F | AGTAACGTCTAGCACTCCGC | |
| GM1-R | TCCAAACTTACACATTGATACACGA | |
| GM2-F | TGGAATCGGAGGGAGGAAAA | |
| GM2-R | GTTGGCAGCCTTCTTTACGC | |
| GM3-F | AACAAGGCTCCATGGCATTAGT | |
| GM3-R | TGGGTTGGTGGGCTTTTTCA | |
| Primers for co-segregation analysis | | |
| PF1 | | TTTGTGGAGCAAAACAGGCAC |
| PR1 | | TCCTTCTTTGGCAAGGGGAAA |
| PF2 | | TTCTGTTCAGTCTGCAAAACACA |
| Primers for complement vector construction | | |
| Rbr7-BamHI-1F | agctcggtacccggggatccAGGGTCGAGAGTTTAGAACA | |
| Rbr7-gene-1R | TTGCATATCATCTAAACTAGCAAAATACTCGTGCATTGCA | |
| Rbr7-gene-2F | TGCAATGCACGAGTATTTTGCTAGTTTAGATGATATGCAA | |
| Rbr7-gene-2R | CCCTCACCAATGCAAACCATAATAAACTGTGAATAGTGGA | |
| Rbr7-gene-3F | TCCACTATTCACAGTTTATTATGGTTTGCATTGGTGAGGG | |
| Rbr7--HindIII-3R | acgacggccagtgccaagcttTTCGGTTTGTCATTACTTT | |
| Primers for subcellular localization vector construction | | |
| pRTVc-RBR7-GFP-F | TGGGATCCCCGGGTGAGCTCATGGCCGCGCACAACACATC | |
| pRTVc-RBR7-GFP-R | GCACTAGTAAGCTTGGTACCTTTCGTTGTCCCTGAAGAAG | |
| Primers for knockout identification | | |
| Rbr7-Crispr-V1-F | AGAACGGGACCTATGTTTGGG | |
| Rbr7-Crispr-V1-R | AAGATGAACAAATCTGACCAACG | |
|  |  | |
| Primers for qPCR | | |
| Q-UBQ-F | AACCAGCTGAGGCCCAAGA | |
| Q-UBQ-R | ACGATTGATTTAACCAGTCCATGA | |
| Q-Rbr7-F | GGTTTGCATTGGTGAGGGTG | |
| Q-Rbr7-R | TCACTAGAATCCGGAGAGGCA | |
| Q-PR10-F | CTCATCCTCGACGGCTACTT | |
| Q-PR10-R | ATCAGGAAGCAGCAATACGG | |
| Q-PR1a-F | CGTCTTCATCACCTGCAACT | |
| Q-PR1a-R | TGTCCATACATGCATAAACACG | |
| Q-PBZ1-F | GGGTGTGGGAAGCACATACA | |
| Q-PBZ1-R | CCTCGAGCACATCCGACTTT | |
| Q-NPR1-F | AACCTGGGTTCTGGTGCAAA | |
| Q-NPR1-R | ACTGTTTTGGAGAGTGCCGT | |
| Q-WRKY19-F | GTCGACGACGGCTACTTCTC | |
| Q-WRKY19-R | CTCGTGGTCCATGTCAACCC | |
| Q-WRKY55-F | CGGAAGCCTATTCTTGGGGG | |
| Q-WRKY55-R | CTGACATTCGGTGCTGCAAG | |
| Q-LOC_Os05g28460-F | GGCATCAACGGACAAGGGAT | |
| Q-LOC_Os05g28460-R | CATCCAAAGTTACCATGTCCAAC | |
| Q-LOC_Os05g28470-F | GGAGCTACCAGTGTCCTCCT | |
| Q-LOC_Os05g28470-R | GCCGATGTTTTGTATACTTGCCT | |
| Q-LOC_Os05g28500-F | GAGGCCTCGGAAACACTCAT | |
| Q-LOC_Os05g28500-R | GGAGAGTCGCTTGCAGAGTT | |
| Q-UBQg-F | TTCTGGTCCTTCCACTTTCAG | |
| Q-UBQg-R | ACGATTGATTTAACCAGTCCATGA | |
| Q-MoPot2-F | ACGACCCGTCTTTACTTATTTGG | |
| Q-MoPot2-R | AAGTAGCGTTGGTTTTGTTGGAT | |
|  |  | |
